# Genome-wide analyses of light-regulated genes in *Aspergillus nidulans* reveal a complex interplay between different photoreceptors and novel photoreceptor functions

**DOI:** 10.1371/journal.pgen.1009845

**Published:** 2021-10-22

**Authors:** Zhenzhong Yu, Christian Streng, Ramon F. Seibeld, Olumuyiwa A. Igbalajobi, Kai Leister, Julian Ingelfinger, Reinhard Fischer

**Affiliations:** 1 Karlsruhe Institute of Technology (KIT)—South Campus, Institute for Applied Biosciences, Department of Microbiology, Karlsruhe, Germany; 2 Nanjing Agricultural University, Key Laboratory of Plant Immunity, Jiangsu Provincial Key Lab for Organic Solid Waste Utilization, National Engineering Research Center for Organic-based Fertilizers, Jiangsu Collaborative Innovation Center for Solid Organic Waste Resource Utilization, Nanjing, China; Oregon State University, UNITED STATES

## Abstract

Fungi sense light of different wavelengths using blue-, green-, and red-light photoreceptors. Blue light sensing requires the “white-collar” proteins with flavin as chromophore, and red light is sensed through phytochrome. Here we analyzed genome-wide gene expression changes caused by short-term, low-light intensity illumination with blue-, red- or far-red light in *Aspergillus nidulans* and found that more than 1100 genes were differentially regulated. The largest number of up- and downregulated genes depended on the phytochrome FphA and the attached HOG pathway. FphA and the white-collar orthologue LreA fulfill activating but also repressing functions under all light conditions and both appear to have roles in the dark. Additionally, we found about 100 genes, which are red-light induced in the absence of phytochrome, suggesting alternative red-light sensing systems. We also found blue-light induced genes in the absence of the blue-light receptor LreA. We present evidence that cryptochrome may be part of this regulatory cue, but that phytochrome is essential for the response. In addition to *in vivo* data showing that FphA is involved in blue-light sensing, we performed spectroscopy of purified phytochrome and show that it responds indeed to blue light.

## Author summary

Fungi are microorganisms with important roles in the environment, as symbionts, as pathogens, or as workhorses in biotechnology. They constantly need to adapt to changing environmental conditions, often far away from their optima. One important environmental factor, fungi respond to is ambient light. The presence of light tells them if they are exposed to a surface and thus potentially to heat, harmful irradiation, or desiccation or other stressful conditions, or whether they are growing inside soil or litter with more constant conditions. Interestingly, many fungi harbor photosensors for blue-, green- and red light. We show here that in the model fungus *Aspergillus nidulans* a large proportion of the genome is under light control, and many genes are regulated through phytochrome and thus by red light. However, phytochrome is also used for blue-light sensing. Many genes are controlled by blue- and by red light signaling systems, but many also respond only to specific wavelengths. The study provides important groundwork for future research to unravel how different genes are regulated at the molecular level and to decipher the biological meaning for the complex light-regulatory systems found in fungi.

## Introduction

Light is a common environmental factor, drives photosynthesis, may cause cellular damage, and may be used for orientation and adaptation. Light sensing is thus very common in plants and animals but not very obvious in microorganisms. However, filamentous fungi and also *Saccharomyces cerevisiae* are able to respond to light. In *S*. *cerevisiae* light is indirectly sensed through the concentration of reactive oxygen species (ROS), which may increase when blue light is absorbed by flavin molecules [1]. This is a basal mechanism probably conserved in many organisms. In comparison, light responses in filamentous fungi may be much more sophisticated and specific, including one or several photoreceptor proteins [2–6]. Only a few of such chromoproteins arose in evolution. Whereas mammals mainly use rhodopsins for light and color sensing, many fungi employ different photoreceptors for blue, green, red and far-red light [7]. Moreover, there are many examples that photoreceptors were multiplied during fungal evolution. Prominent examples with large numbers of putative photoreceptor proteins are *Phycomyces blakesleanus*, *Mucor circinelloides*, *Botrytis cinerea* or *Knufia petricola* [3,8–12]. This makes fungi especially attractive to study the role of individual photoreceptors but also their functional interplay and perhaps also their evolution.

At the molecular level, blue-light responses have been studied best in *Neurospora crassa* where they are connected to the circadian clock [13–15]. Although the phenomenon had been described decades ago and blue-light response mutants were isolated (white collar 1, 2), it took until 2002 that the photoreceptor (WC-1) was discovered. Interestingly, it was a transcriptional regulator with a bound flavin molecule [16,17]. Therefore, an elaborate signal transduction cascade is not required to transmit the light signal to differential gene expression. Nevertheless, in some fungi the blue-light response is interlinked with other signaling cascades [18]. *N*. *crassa* genome-wide expression analyses revealed that a large proportion (up to 30%) of the genome is under light control [19–21]. In *Trichoderma atroviride* similar numbers of differentially-regulated genes were obtained [22]. Besides the dominating blue-light response, *N*. *crassa* is also able to respond to red light using phytochrome and to green light with the help of opsins although their contribution at a genome-wide level is rather marginal [23–25].

A quite different light-response appears to be realized in *Aspergillus nidulans*, which shows a pronounced red-light response with phytochrome being the main photoreceptor protein [26,27]. The red-light response has been studied best in *A*. *nidulans*, where phytochrome is the photoreceptor, and the signal is transmitted from the cytoplasm using the HOG MAP kinase pathway into the nuclei. In addition, a fraction of phytochrome acts in nuclei on chromatin remodeling enzymes [4,28–31]. Another important regulator of the light response in *A*. *nidulans* is the Velvet protein, VeA [32–34]. It is likely to be a transcriptional activator and functionally and physically interacts with phytochrome [27,35]. In addition to phytochrome, *A*. *nidulans* harbors orthologues of the *N*. *crassa* white collar proteins, although they appear to have repressing functions [27]. Another example for a fungus with a pronounced red-light photo-response is *Alternaria alternata* [36]. Recently, two hemeoxygenases were identified, which are involved in phytochrome chromophore biosynthesis and defined a novel role for fungal mitochondria [37]. In addition to red- and blue-light photoreceptors, *A*. *alternata* harbors two putative opsins as green-light stimulated proton pumps.

Many of the conclusions on the mechanism of phytochrome-dependent light sensing in *A*. *nidulans* are based on expression analyses of two light-induced genes, *ccgA* and *conJ* [4]. Whether those results can be generalized to all red-light or all light-induced genes, remained an open question. Previous genome-wide expression analyses using microarray approaches revealed that a large proportion of the genome is under light control, but it remained unclear if different photoreceptors control distinct classes of genes [19,22,38]. It was also shown that red light stimulates conidiation and the effect could be reversed by far-red light illumination [39]. On the other hand far-red light inhibited germination stronger than red light, with phytochrome being required for the response [40]. There was also evidence that phytochrome is required for the blue-light dependent phosphorylation of the HOG MAP kinase SakA [31]. Here we show that in *A*. *nidulans* more than 1100 genes (ca. 10% of the genome) are differentially expressed after 15 min of illumination and that there are commonly regulated genes as well as specific classes of red-, far-red and blue-light controlled genes. In addition, we show novel functions for phytochrome in far-red light and in blue-light sensing.

## Material and methods

### Strains, plasmids and culture conditions

Minimal medium (MM) for *A*. *nidulans* was prepared as described [41]. *A*. *nidulans* strains used in this study are listed in **Table 1**.

**Table 1:** Strains used in this study.

Name	Genotype	Source
FGSCA1153	*yA1*, *pabaA1; argB2; pyroA4*, *nkuA*::*bar*	Fungal Genetics Stock Center
SKV103	*pyrG89; pyroA4; veA* ^+^	[57]
SJP1	*pyrG89; ΔargB*::*trpCΔB; pyroA4; ΔfphA*::*argB; veA*^*+*^	[27]
SJP21.3	*pyrG89; ΔlreB*::*argB; ΔargB*::*trpCΔB; pyroA4; ΔlreA*::*argB; ΔfphA*::*argB; veA*^*+*^	[27]
SJR2	*pyrG89; pyroA4*, *nkuA*::*bar; veA*^+^	[28]
SJR10	*pyrG89*; *ΔargB*::*trpCΔB*; *pyroA4*, *nkuA*::*bar; ΔfphA*::*argB*; *veA*^+^	[28]
SSR66	*yA2*, *pabaA1; pyroA4*, *nku*::*bar; ΔlreA*::*ptrA; veA+*	[28]
SZY31	*pyrG89; ΔsakA*::*AfriboB; pyroA4*, *ΔnkuA*::*argB; veA*^*+*^	[31]
SJR6	FGSC A1153 x SKV103: *yA1*, *pabaA1; argB2; pyroA4*, *nkuA*::*bar; veA*^*+*^	J. Rodriguez, KIT
SJR18	SJR6 transformed with the plasmid pJR18-*conJ(p)*::*lucI*::*argB*: *yA1*, *pabaA1; argB2*, *conJ(p)*::*lucI*::*argB; pyroA4*, *nkuA*::*bar; veA*^*+*^	J. Rodriguez, KIT
SJR21	SJR6 transformed with the plasmid pJR19-*ccgA(p)*::*lucI*::*argB*: *yA1*, *pabaA1; argB2*, *ccgA(p)*::*lucI*::*argB; pyroA4*, *nkuA*::*bar; veA*^*+*^	J. Rodriguez, KIT
SJR28	SJP21.3 x SJR18: *conJ(p)*::*lucI*, *pyroA4*, *pabaA*, *ΔlreA*::*argB; veA*^*+*^	J. Rodriguez, KIT
SJR36	SJP21.3 x SJR21: *ccgA(p)*::*lucI*, *pyroA4*, *pabaA*, *ΔlreA*::*argB; veA*^*+*^	J. Rodriguez, KIT
SJR40	SJR18 x SJP1: *conJ(p)*::*lucI*, *pyroA4*, *pabaA*, *ΔfphA*::*argB; veA*^*+*^	J. Rodriguez, KIT
SJR41	SJR21 x SJP1: *ccgA(p)*::*lucI*, *pyroA4*, *pabaA*, *ΔfphA*::*argB; veA*^*+*^	J. Rodriguez, KIT
SCK44	*yA1*, *pabaA1; argB2; pyroA4*, *nkuA*::*bar*, *cryA*::*ptrA*	[40]

*E*. *coli* BL21 (DE3) was used for heterologous expression of recombinant *A*. *nidulans* FphA. An *E*. *coli* K12 codon optimized synthetic version of *A*. *nidulans fphA* was cloned into the pASK-IBA3 vector (IBA, Goettingen) [42]. BphO_pACYCDuet was used for biliverdin production.

### Light boxes

The panel (printed circuit board) measures 39 cm in width and 28 cm in length. Onto the panel 12 monochromatic LEDs of a wavelength of 450 nm (blue, SMBB450H-1100, Ushio), 525 nm (green, SMBB525V-1100 rev. B, Ushio), 700 nm (red, SMBB700-1100, Ushio) and 760 nm (far red, SMBB760-1100, Ushio) with a tolerance of +/- 10 nm and 12 5600 K white LEDs (Q65112-A1637, Osram) were soldered in 12 clusters consisting of one LED of each type with the idea in mind to illuminate up to 12 Ø 92 mm petri dishes. A self-made software was coded for Photon P1 WIFI module in order to address intensities of each light type and specific timings. To calibrate the software, an ocean optics JAZA0503 spectrometer was used. For each LED type the photon flux was measured at a distance of 20 cm. The photon flux in following experiments referred to this calibration. For safe operation, the maximum photon flux was limited to 17 μmol photons m^-2^ s^-1^. Homogeneous illumination was ensured by 130° viewing angle for the monochromatic and 120° for the white LEDs and placing the sample centered under the LED cluster.

Lightboxes were calibrated by absolute intensity measurements using a portable JAZ-COMBO S/N:JAZA0503 with QP400-1-VIS-NIR and CC-3-UV-S spectrometer unit (Ocean Optics). The measurements were evaluated using the complementary SpectraSuite software and integrating the area below the curve to determine the absolute intensity in μmol photons per square meter and second (μmol photons m^-2^ s^-1^). The necessary LED power to reach the desired intensities was individually determined for each type of LED.

### Luciferase-based reporter assay

In order to facilitate the quantification and comparison of light responses, we developed a luciferase-based reporter assay. To this end the promoter of interest (*ccgA*, *conJ*) was cloned in front of the luciferase gene in a plasmid containing an *argB* mutant allele. The plasmid was used to transform an *argB2* mutant in which a deletion of a single cytosine nucleotide at position 248 from the start codon results in a stop codon at codon 115. Integration of the circular *argB* containing plasmid *in locus* after the *argB2* mutation, restores the functional *argB*-open reading frame. The reporter construct was then further introduced into photoreceptor mutants by crossing. This strategy of *in locus* integration guarantees comparability of the results between different strains.

The luciferase reporter plasmids (pJR18 *conJ(p)*::*lucI*::*argB*, pJR19 *ccgA(p)*::*lucI*::*argB*) with the *conJ* or the *ccgA* promoter (1 kb) were constructed using the following oligonucleotides: Luc-BamHI-for CGC AGG ATC CAT GGA GGA CGC CAA GAA CAT C; Luc-EcoRI-rev GCC GGA ATT CAG AGC TTG GAC TTG CCG CC; pccgA-XhoI CTT CTA CCT CGA GCC TCA AAC AAT TCG ACG; pccgA-BamHI GCG CTG GGA TCC TGC GAT TGT TTA TAT G; pconJ-XhoI CTT CTA CCT CGA GAT GGT CTT ACA GTC GAC; pconJ-BamHI GCG CTG GGA TCC TTG ATG TAT TTA AAG AAT TG.

For the luciferase-based reporter assay, each well of a 96 well plate was filled with 170 μl MM, supplemented with 1 μM D-luciferin (Perkin Elmer). The plate was incubated at 37°C for 16 h in the dark. After the incubation period, samples were measured with a plate reader (Perkin Elmer Enspire Multimode) every 10 min repeatedly for 90 min. The plate was illuminated outside the plate reader under the panel. As soon as the measurement started, the plate was pulled into the machine and the gate at the front closed. Hence, measurements of luciferase were performed in darkness, but as soon as the measurement was done, the plate was illuminated again. For the illumination with blue light, we used the same LED panels as described above.

### Analysis of reactive oxygen (ROS) concentrations

Simulation of the lightbox settings for ROS measurements was done using a combination of the Zeiss Axiomanager Z1 (Carl Zeiss, Jena) halogen lamp and blue SCHOTT GLAS optical filters (Type: BG28, 2 mm thickness). Voltage settings were determined with the same portable JAZ-COMBO spectrometer system used for lightbox calibration. The focus was set while using a green filter to prevent activation of *A*. *nidulans* light responses. ROS measurements were performed using the CellROX Orange Reagent (Invitrogen, Thermo Fisher Scientific). Precultures of the respective strains were grown in the dark on cover slips over night at room temperature. Afterwards, cells were incubated with 2 μmol of CellROX Orange reagent in minimal medium (with the appropriate supplements) in the dark for 25 min. Then, cells were washed twice with PBS for 5 min each. Cover slips were then flushed once with minimal medium and mounted on microscope slides covered with a thin layer of solid medium (1% agarose). At times when some visibility was required during preparation, green light (approx. 520 nm) was used to prevent activation of the *A*. *nidulans* light responses and premature induction of reactive oxygen species. Time-lapses were recorded for 30 min with one picture taken every 20 seconds. ROS signals were quantified with ImageJ. Values were normalized to the greyscale value of the initial picture in each time-lapse. As small increases of background fluorescence were detected in some time-lapses, the background signal was also recorded and subtracted.

### RNA extraction and reverse transcriptase-quantitative PCR (RT-qPCR)

Conidia of different strains were inoculated on the surface of liquid minimal medium (2% glucose) supplemented with 1 μg/ml pyridoxine HCl, 1 mg/ml uracil, 1 mg/ml uridine and 1 μg/ml p-amino benzoic acid and cultured in the dark for 18 hours at 37°C. Afterwards, red-, blue- and far-red light was imposed separately for 15 min. Double treatment with two different light qualities was performed by illumination for 15 min with the first wavelength, followed by 15 min illumination with the second. The mycelia were collected and frozen in liquid nitrogen. To analyze the gene expression in response to oxidative stress, the strains were cultured in liquid medium (2% glucose plus supplements) in shaking flasks at 37°C for 24 hours and then 30 mM hydrogen peroxide was imposed for 15 min or 30 min. RNA was extracted (E.Z.N.A. Fungal RNA Mini Kit, Omega Bio-Tek) and DNA digested (TURBO DNase, Invitrogen). Expression of the light regulated genes *ccgA* and *ccgB* was measured by reverse transcriptase-quantitative PCR (RT-qPCR) (SensiFAST SYBR No-ROX One-Step Kit, bioline) with the *h2b* gene as a control. Primers used were: h2b-RT-F (5’-CTG CCG AGA AGA AGC CTA GCA C-3’), h2b-RT-R (5’-GAA GAG TAG GTC TCC TTC CTG GTC-3’), ccgA-RT-F (5’-CGA CGC TTC CCT CAC TTC TC-3’), ccgA-RT-R (5’-CAT CAT GGG ACT TCT CGT CCT T-3’), ccgB-RT-F (5’-GGA GACT ATC AAG GTA AGC ATG TAC C-3’), ccgB-RT-R (5’-CTT GTC AAA GAG AGC GTC CTT G-3’).

### RNA sequencing

Fresh conidia of wild type and the mutants (40 μl conidia suspension with 1x10^8^ conidia/ml) were inoculated on the surface of 3 ml supplemented liquid minimal medium (2% glucose, plus appropriate supplements) in petri dishes (3 cm diameter, 52 petri dishes in total) and cultured in the dark for 18 hours at 37°C. Afterwards, samples were exposed to red-, blue- and far-red light separately or kept in the dark (dark control) for 15 min. Mycelia were harvested in dim green light immediately after light exposure and frozen in liquid nitrogen. For each condition we prepared four biological replicates (in the following experiment, three of them with better quality were chosen for RNA sequencing). Cell disruption was performed with a cell homogenizer (MM200, Retsch) at 30 hits/min for 5 min and RNA was extracted with the RNeasy plant mini kit (Qiagen). RNA samples were treated with the TURBO DNA-free kit (AM1907, Invitrogen, Thermo Fisher Scientific). The quality of the RNA samples was analyzed with the Agilent 2100 Bio analyzer (Agilent RNA 6000 Nano Kit). The RNA integrity number (RIN) of each sample was higher than 9.5 and the ratios of 28S/18S rRNA of the samples were between 2.4 and 3.6. The ratio of A260/A280 was more than 2.0 and A260/A230 was more than 1.8.

To construct the library, mRNA of three biological replicates of each condition was enriched by oligo dT selection. The library was constructed by BGI GENOMICS Co., Ltd. using *in house* reagents. RNA samples were fragmented and reverse-transcribed to double-strand cDNA using N6 random primers. The synthesized cDNA was subjected to end-repair and then 3’ adenylated and further ligated with adapters. PCR amplification was performed to enrich cDNA templates. The PCR product was denatured by heat and then single strand DNA was cyclized with splint oligo and DNA ligase. The samples were sequenced on the BGISEQ-500 platform at BGI (BGI GENOMICS Co., Ltd.).

The clean reads were filtered using BGI internal software SOAPnuke (version: v1.5.2, https://github.com/BGI-flexlab/SOAPnuke) from the low-quality reads, reads with adaptors and reads with unknown bases and were stored in FASTQ format [43]. The clean reads were mapped to the reference genome (http://www.aspgd.org) using Bowtie2 (version: v2.2.5, http://www.ccb.jhu.edu/software/hisat) [44]. Gene expression levels were calculated with RSEM (version: v1.2.12, http://deweylab.biostat.wisc.edu/RSEM ) [45]. Differentially expressed genes (DEGs) (fold change ≥ 2, adjusted p-value ≤ 0.05) were identified with the DEseq2 algorithm [46]. DEGs were classified according to official classification (KEGG). With the GO and KEGG annotation results, GO functional and KEGG pathway enrichments were performed with the OmicShare tools (https://www.omicshare.com/tools/). Heatmaps were generated in R environment.

### Spectral analysis of phytochrome

Heterologous expression of FphA from *A*. *nidulans* was carried out using a *tet* promoter driven expression system under the control of an anhydrotetracycline (AHT) inducible promoter (IBA, Goettingen). The expressed protein contains a C-terminal *Strep-tag*. *E*. *coli* BL21 (DE3) cells were co-transformed with a plasmid coding the heme oxygenase BphO from *Pseudomonas aeruginosa* and the FphA coding plasmid. Holo FphA was expressed and purified in dim green light as described previously [26,42].

Spectra were recorded on a JASCO V-750 spectrophotometer. All experiments were performed under green safelight. Sample irradiation was carried out manually using a custom-built lightbox with appropriate irradiation devices of blue light (450 nm, 6.8 μmol m^-2^ s^-1^, 30 min irradiation), red light (700 nm, 3.4 μmol m^-2^ s^-1^, 2 min irradiation) and far-red light (760 nm, 3.4 μmol m^-2^ s^-1^, 4 min irradiation).

## Results

### Reactive oxygen species (ROS)-versus photoreceptor mediated light responses

**Fig 1:**
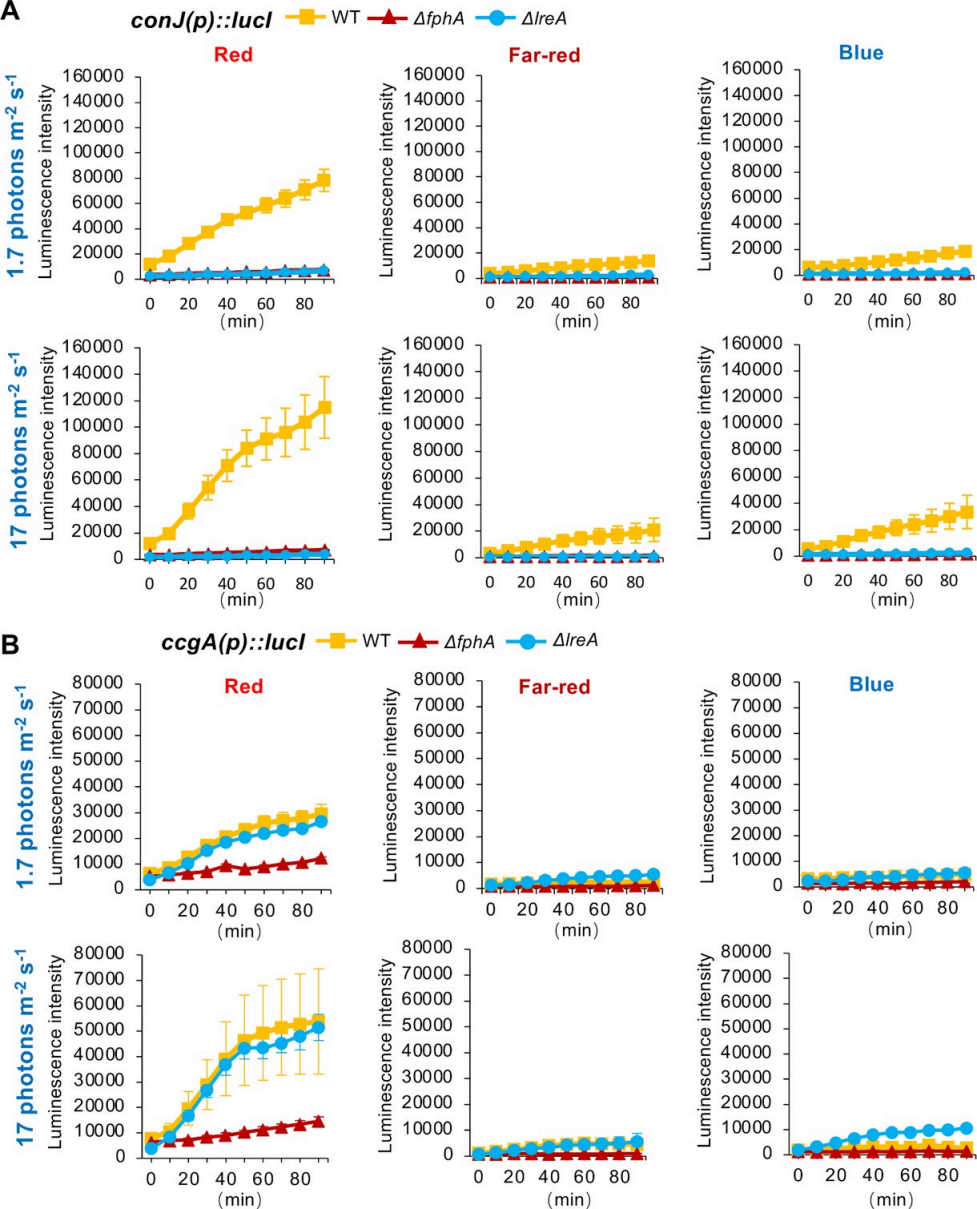
Luciferase-based reporter assay. **(A)** Luciferase was cloned behind the *conJ* promoter and transformed into wildtype, the *ΔfphA-* or the *ΔlreA-*deletion strains. After 16 h incubation in the dark, luminescence was measured every 10 min for 90 min under the indicated light conditions. **(B)** Measurement was performed like in **(A)**, but the luciferase was under the control of the *ccgA* promoter. The graphs represent the mean of three biological replicates with two or three technical replicates. Error bars indicate standard deviation.

Light may interfere with different cellular processes through the generation of reactive oxygen species (ROS), which in turn can cause drastic changes in gene expression [1]. Because we were interested in specific, photoreceptor-dependent light responses, we first tested the expression of two previously known light-induced genes, *ccgA* and *conJ* to optimize the illumination conditions and reduce any side-effects by ROS before the global expression analyses [28]. To this end, LED panels were designed which allowed easy changes of the light intensities or the use of specific wavelengths. Measurement of the spectrum of the emitted light of the LEDs confirmed peaks at 450 nm for blue light, 700 nm for red light and 760 nm for far-red light **(S1A Fig)**. We also developed a luciferase-based reporter system to follow light-induced gene induction. The luciferase construct was integrated at the *argB* locus in all strains to guarantee comparability of the results. Strains were incubated in a 96 well plate (mounted in the plate reader) filled with minimal medium (MM) containing 1 μM luciferin for 16 h at 37°C in the dark. Luminescence was low and remained under a value of 10.000 when kept in the dark. During illumination with two different intensities (1.7 and 17 μmol photons m^-2^ s^-1^), samples were measured repeatedly every 10 min for 90 min **(Fig 1A and 1B)**. Different background luminescence in the different samples before illumination are probably due to the different strains, because same amounts of conidia were used for inoculation. As described earlier, both genes responded well to red light and light-induction required phytochrome [28]. Photoadaptation, as it is observed when followed RNA levels **(S1B Fig)** was not observed, probably because luciferase is a quite stable enzyme. The red-light response of *conJ* required FphA and also the blue-light receptor LreA whereas *ccgA* was also induced in the absence of LreA. **(Fig 1A and 1B)**. Both genes were also induced by far-red and by blue light, although to much lower extents. Far-red and blue-light induction of *conJ* required FphA and LreA. In the response of *ccgA*, LreA appeared to have a repressing function. This was most obvious in blue light of higher intensity **(S2 Fig)**.

Taken together we provide first evidence that FphA plays a role in blue light sensing, and that, as shown before [28], LreA can be an activator but also a repressor. In addition, red light, far-red and blue light can stimulate gene expression, but different genes appear to respond differently. We observed that blue-light stimulated *ccgA* expression was independent of LreA at low intensities.

**Fig 2:**
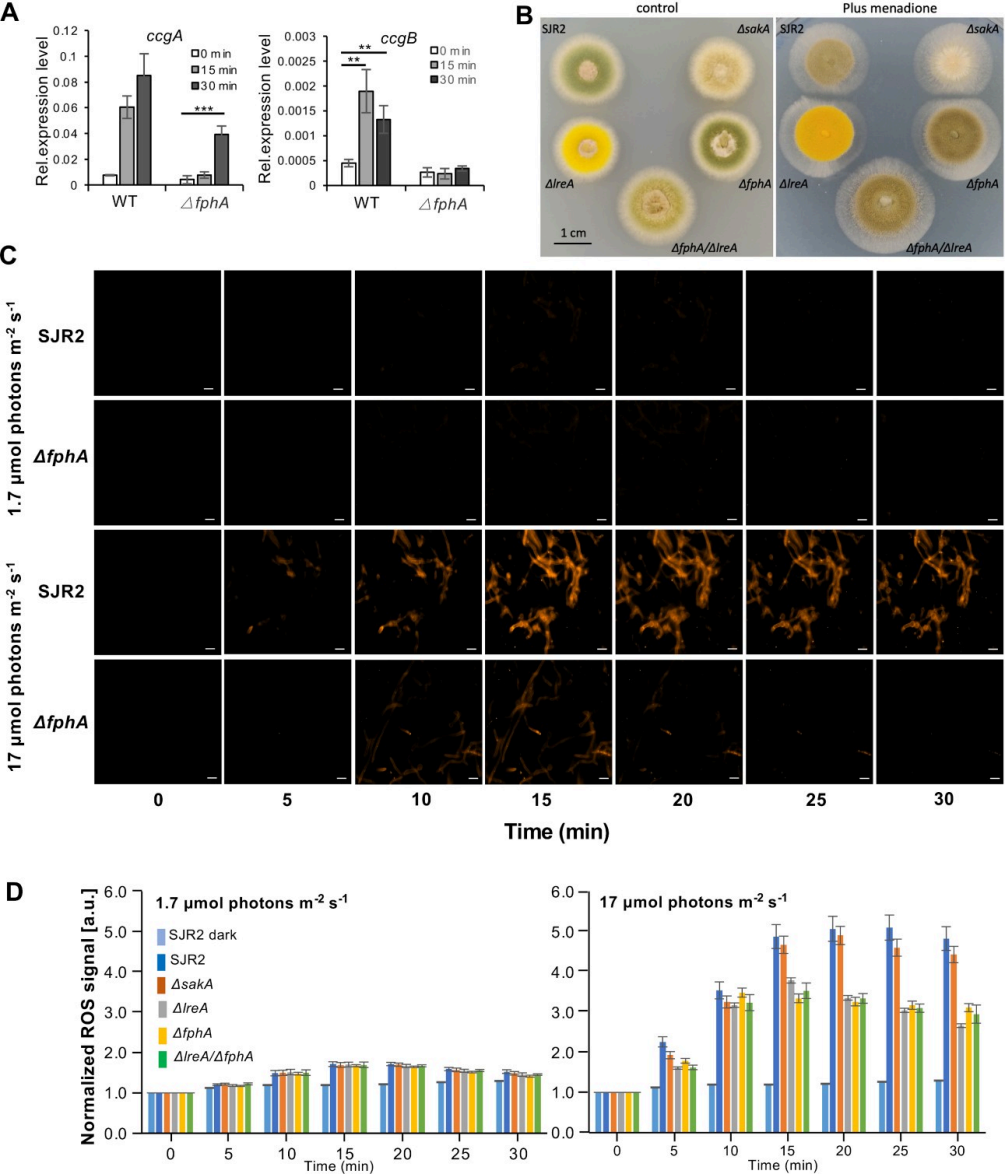
Reactive oxygen species (ROS) levels in wild type (SJR2) and the *ΔfphA-*, the *ΔlreA-*, the *ΔsakA-* and the *ΔfphA*/*ΔlreA*-deletion strains. **(A)** Expression analysis of *ccgA* and *ccgB* under oxidative stress. The mycelia grown in supplemented liquid minimal medium in shaking flasks in the dark were harvested after 30 mM hydrogen peroxide was imposed for 15 min and 30 min. The expression of *ccgA* and *conJ* was normalized to the *h2b* gene. The error bar was calculated from three biological replicates. Significant differences were calculated using the two-sample *t*-test (**P<0.01 and ***P<0.001). **(B)** Growth of indicated strains on minimal medium (control) and minimal medium containing 100 μM menadione at 37°C. 5000 spores were inoculated for each strain, and pictures were taken after 48 hours (control) and 120 hours (minimal medium + 100 μM menadione. **(C)** Representative images of the ROS signals quantified in **(D)**. The WT and *ΔfphA*-strains were chosen as examples. Scale bar = 10 μm. **(D)** Quantification of ROS signals after blue-light illumination at 1.7 μmol photons m^-2^ s^-1^ (left) and at 17 μmol photons m^-2^ s^-1^ (right). Relative increase of ROS signals compared to the initial value (normalized to 1). Error bars indicate standard deviation.

We next tested, if the light responses were specific for light or could be caused indirectly through reactive oxygen species (ROS). We have chosen *ccgA* and *ccgB*, two genes which respond well to blue light, for this analysis, because blue-light induction of *conJ* was not very pronounced, and the error bars were always quite large. Both genes, *ccgA* and *ccgB* were indeed induced not only by light but also after incubation of the mycelium in the presence of 30 mM H_2_O_2_ for 15 or 30 min **(Fig 2A)**. There was also evidence in *A*. *fumigatus* and in *A*. *alternata* that phytochrome mutants were more resistant towards oxidative stress [37,47]. Therefore, we tested the growth of WT and different mutant strains on minimal medium containing menadione (100 μM). Indeed, all photoreceptor mutants produced larger colonies than wild type **(Fig 2B)**. The concentration of intracellular ROS was then monitored microscopically using the CellROX Orange reagent (Invitrogen; Thermo Fischer Scientific). The membrane diffusible reagent absorbs light of 545 nm and emits light of 565 nm after oxidation. Upon illumination with blue light at an intensity of 1.7 μmol photons m^-2^ s^-1^, fluorescence was very weak. When the intensity was raised to 17 μmol photons m^-2^ s^-1^, the fluorescence intensity increased in all hyphae, indicating ROS generation in response to intense blue light **(Fig 2C and 2D and S1 Movie)**. Other wavelengths (green—approx. 530 nm; red—approx. 680 nm; far-red—approx. 720 nm) did not result in an increase of the intracellular ROS signal neither at low nor at high intensity. We next analyzed the contribution of FphA, LreA and the stress activated MAP kinase, SakA in the high-intensity blue-light response. In all strains the concentration of ROS increased similarly for ca. 15 min. Whereas the ROS concentration in wild type and the *ΔsakA*-deletion strain further increased for ca. 5 min, the concentration remained at a plateau in the *ΔlreA*-deletion strain. In the *ΔfphA*-deletion strain the concentration also reached a plateau but slightly lower than in the *ΔlreA* mutant. It appears that the *ΔfphA*-deletion strain is more resistant to oxidative stress.

Taken together, the results still show that ROS generation occurred at 17 μmol photons m^-2^ s^-1^, which induced *ccgA* to some extent, but with 1.7 μmol photons m^-2^ s^-1^ ROS generation was very low and *ccgA* expression was not stimulated. From this we anticipate that low-intensity light conditions should not contribute significantly to gene activation.

To unravel the relevance of these findings with regards to whole genome expression control, we performed RNAseq analyses under low-light intensity conditions. Because phytochrome hubs into the SakA MAP kinase pathway, we expected a strong effect of illumination on stress-related genes and included the *ΔsakA*-MAP-kinase deletion strain in the expression profiling experiments.

### Different wavelengths control common but also specific sets of genes

Wild type (SJR2) was illuminated with 1.7 μmol photons m^-2^ s^-1^ for 15 mins after 18 hours culture in the dark on the surface of supplemented minimal medium. We have chosen 15 min of illumination because longer illumination caused a reduction of the *ccgA* and *conJ* expression levels due to adaptation processes **(S1B Fig)**. In addition, the relative short exposure time should eliminate secondary effects after light control of key regulators for e.g. development or secondary metabolism. The temperature in the incubation chambers was documented and did not rise due to the illumination. In *N*. *crassa* it was shown that the white collar proteins are recruited to a blue-light controlled promoter after 15 min [19]. In *A*. *nidulans* LreA binds to the *ccgA* promoter in the dark and is released 15 min after blue-light illumination [28].

**Fig 3:**
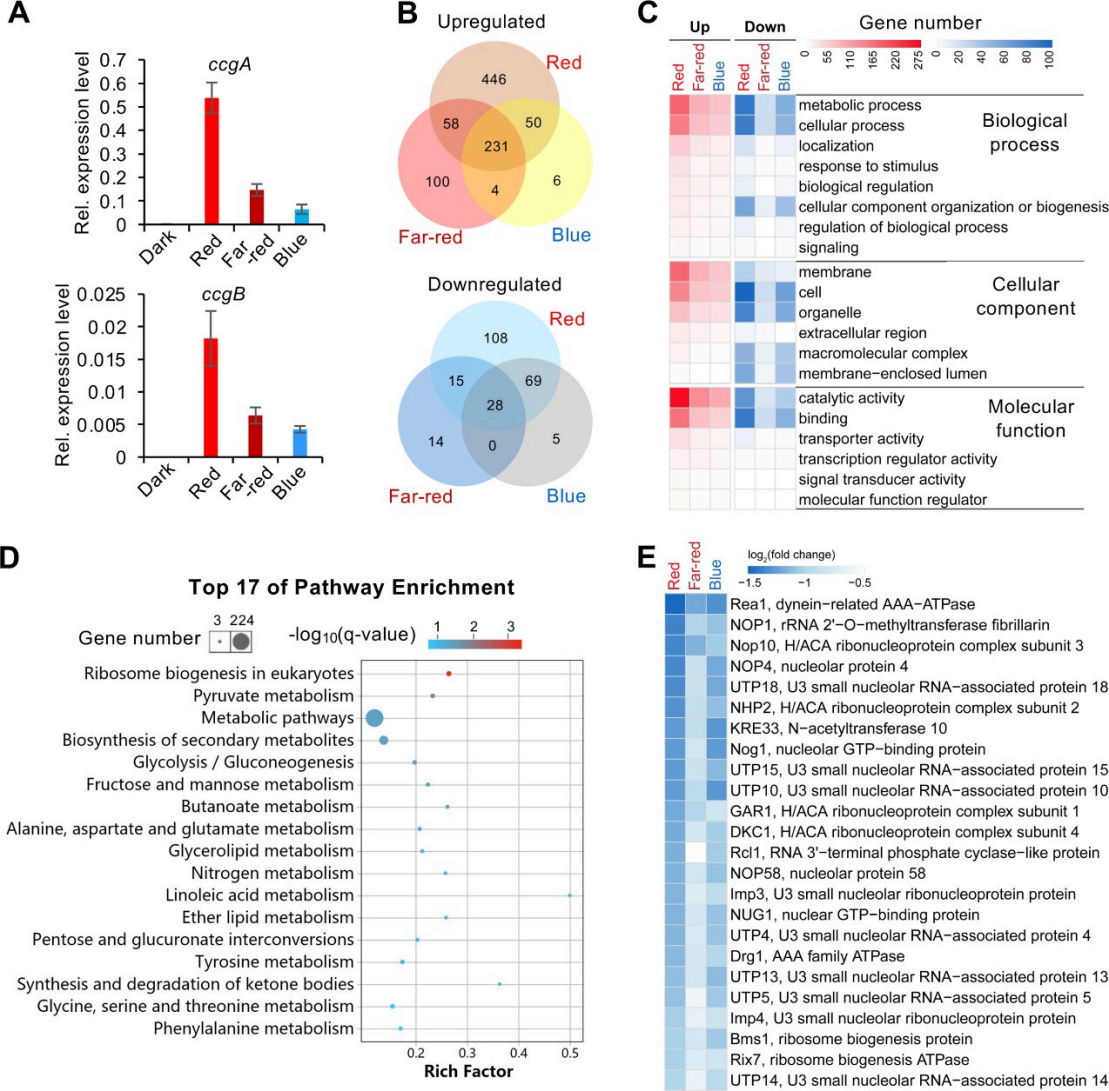
Transcriptional response of the wild-type strain upon red, blue and far-red light exposure. **(A)** Expression of *ccgA* and *ccgB* in wild type in red-, blue- and far-red light quantified by reverse transcriptase-quantitative PCR (RT-qPCR). Red, blue and far-red light with the intensity of 1.7 μmol photons m^-2^ s^-1^ was applied for 15 min before RNA isolation. The expression of *ccgA* and *conJ* was normalized to the *h2b* gene. The error bar was calculated from three biological replicates. **(B)** Venn diagram analysis of the differentially expressed genes (DEGs) (fold change ≥ 2, adjusted p-value ≤ 0.05) identified in red, far-red and blue light. **(C)** GO enrichment analysis of DEGs. **(D)** KEGG pathway analysis performed with all DEGs identified in red, far-red and blue light (p-value < 0.05). The color of the bubbles indicates the value of -log_10_(q-value) and the q-value represents the corrected p-value. Rich factor refers to the quotient of the number of DEGs and the total gene number in the pathway. **(E)** List of DEGs involved in ribosome biogenesis. Heatmap displays values of log_2_(fold change) of DEGs. Color scale, -1.5 ≤log_2_(fold change) ≤0.5.

Before the RNAseq experiment, we validated the extracted RNA by checking the expression of two light-induced genes, which were the top candiates from a previous expression analysis [38]. Both were induced by red-, far-red and by blue light **(Fig 3A)**. In total 1134 genes were differentially expressed (DEGs) (fold change ≥ 2, adjusted p-value ≤ 0.05) after red, far-red or blue light exposure as compared to dark incubation **(S1 Table)**. 895 genes were up- and 239 genes were downregulated. The largest change in the expression level was observed under red-light conditions, where 785 genes were upregulated and 220 downregulated. Under far-red light, 393 genes were up- and 57 downregulated. Blue light had the least effect with 291 up- and 102 downregulated genes **(Fig 3B)**. Among the upregulated genes, 231 genes were upregulated by all three wavelengths, whereas 446 genes were red-light, 100 genes were far-red-light, and only 6 genes were blue-light specific. Notably, 97% (281 out of 291) of blue-light inducible genes were also induced by red light, and 81% (235 out of 291) were also induced by far-red light. Among the light-repressed genes, 220, 57, and 102 genes were repressed by red, far-red, and blue light, respectively. 108 genes were red-light specific, 14 genes were far-red light, and five genes were blue-light specific. 95% of blue-light repressed genes were also repressed by red light.

Next, DEGs were analyzed for their functions using Gene Ontology (GO) **(Fig 3C and S2 Table)**. The “Top one” enriched GO-term of genes upregulated in red, far-red and blue light is *catalytic activity*, followed by *metabolic process* and *membrane*. Among the downregulated genes, the GO-terms *cell* and *cell part* enriched most significantly, followed by *metabolic process*. KEGG pathway analysis was further performed with all DEGs, and the “Top 17” enriched pathways (p value < 0.05) are listed **(Fig 3D and S3 Table)**. Interestingly, ribosome biogenesis was the first category, and all 24 ribosome biogenesis genes were repressed by red, far-red and blue light **(Figs 3E and S3)**. These results show massive changes of the transcript abundance of metabolic genes, and the down regulation of ribosome biogenesis may point to a delay of translation and thereby a delay of changes of the metabolism. Likewise, in *N*. *crassa* light repressed the rRNA metabolism [21].

### Phytochrome is the dominant photoreceptor for red- and blue-light responses

**Fig 4:**
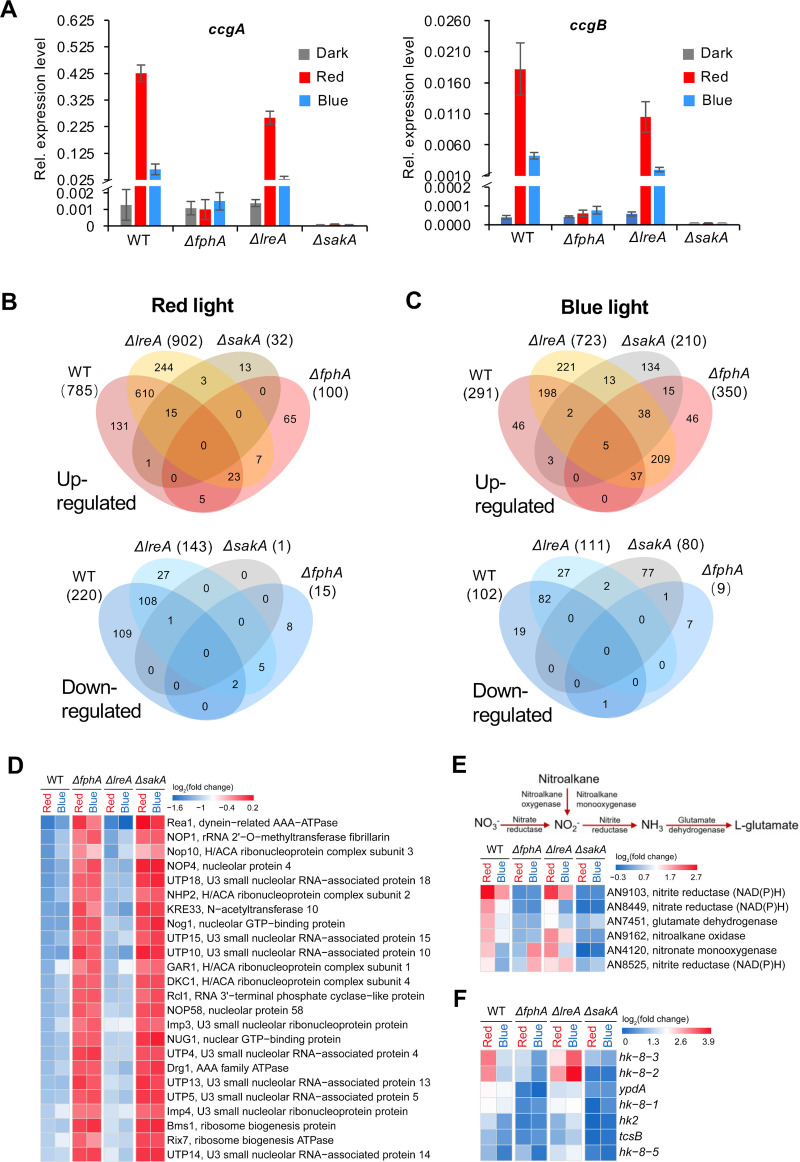
Transcriptional response of wild type, the *ΔfphA*-, the *ΔlreA*- and the *ΔsakA*-deletion strains upon red, blue and far-red light exposure. **(A)** Expression of *ccgA* and *ccgB* in different strains in red-, blue- and far-red light measured by reverse transcriptase-quantitative PCR (RT-qPCR). The experiment was performed as above. The error bar was calculated from three biological replicates. **(B)** Venn diagram analysis of DEGs identified in different strains upon red light illumination. **(C)** Venn diagram analysis of DEGs identified in different strains upon blue light exposure. **(D)** Heatmap of the DEGs involved in ribosome biogenesis in the strains indicated. The colors of heatmap represent log_2_(fold change) of DEGs. Color scale, -1.6≤log_2_(fold change)≤0.2. **(E)** Heatmap of the DEGs involved in nitrogen assimilation in the strains indicated. Heatmap displays log_2_(fold change) of DEGs. Color scale, -0.3 ≤log_2_(fold change)≤2.7. **(F)** Heatmap of the DEGs involved in two component regulatory systems. Heatmap displays log_2_(fold change) of DEGs. Color scale, 0 ≤log_2_(fold change) ≤3.9.

After the analyses of the DEGs, we were interested in the mechanisms explaining the observed transcriptional changes after 15 min of illumination and compared transcriptional changes in wild type (SJR2), a *ΔfphA-* (SJR10), a *ΔlreA*- (SSR66) and a *ΔsakA*-deletion strain (SZY31). All strains contained the wild-type allele of the *velvet* gene (*veA^+^*). In order to link the new data using the new illumination system to previously obtained data we first analyzed the expression of the two light-regulated genes *ccgA* and *ccgB*. In the *ΔfphA*-deletion strain *ccgA* and *ccgB* did not respond to light, but in the *ΔlreA*-deletion strain these two genes were induced well in red and to some extent even in blue light. In the *ΔsakA*-deletion strain, the expression of *ccgA* and *ccgB* was downregulated in the dark in comparison to wild type and both genes were not induced by light anymore **(Fig 4A)**. These results can be explained with the current model that phytochrome signals through the HOG pathway and that LreA can have a repressing function [4,28,31]. Next, we studied the role of FphA, LreA and SakA in the regulation of all light regulated genes. Therefore, a hierarchical clustering analysis of all DEGs identified in wild type, a *ΔfphA-*, a *ΔlreA-*, and a *ΔsakA*-deletion strain upon red and blue light exposure. In the heatmap of the hierarchical clustering DEGs of wild type in red light and of the *ΔlreA-*deletion strain in both red and blue light were clustered in group I and the others fell into group II **(S4 Fig)**.

Five expression profiles of the *ΔlreA*-deletion strain in blue light, the *ΔfphA*-deletion strain and the *ΔsakA*-deletion strain in blue and red light respectively were clustered into group II **(S4 Fig)**. The *ΔfphA*-deletion strain and the *ΔsakA*-deletion strain in blue and red light respectively were clustered into the subgroup of group II, where less genes were differentially expressed. 96% (757 out of 785) of red-light inducible genes detected in wild type were not induced anymore by red light in the absence of phytochrome **(Fig 4B)**. However, there were still 100 genes induced in the *ΔfphA*-deletion strain by red light, 72 of which were not induced in wild type. The expression of one of such genes was validated by quantitative real time PCR **(S5A Fig)**.

In blue light, 350 genes were induced in the *ΔfphA*-deletion strain, but only 42 of those genes were blue-light induced in wild type **(Fig 4C)**. Interestingly, when *fphA* was deleted, a set of light-inducible genes, which were not induced in wild type by red- (72 genes) or blue light (308 genes), were induced. 86% (249 out of 291) of blue-light inducible genes detected in wild type were not induced in the absence of FphA. An example of a gene which was still induced in blue light without FphA was validated by RT-qPCR **(S5B Fig)**.

In cluster 2, most of the genes that were repressed by red- and blue light could not be repressed anymore in the *ΔfphA*-deletion strain **(S4 Fig)**. In wild type 220 genes were repressed by red light, but 218 of them (99%) could not be repressed anymore without FphA **(Fig 4B)**. Likewise, 101 genes out of 102 (99%) repressed by blue light in wild type were not detected in the *ΔfphA*-deletion strain **(Fig 4C)**. Hence, phytochrome plays a key role in light-dependent gene repression.

### LreA represses light responses in blue- and in red light

To our surprise, the *ΔlreA*-deletion strain appeared to be more sensitive to both red and blue light. Most of the upregulated genes in group I had higher expression level in the *ΔlreA*-deletion strain in red light. More DEGs were identified in the *ΔlreA*-deletion strain in comparison to wild type in red- and blue light respectively. In the *ΔlreA-*deletion strain, 902 genes were upregulated in comparison to 785 genes in wild type in red light and, moreover, most of the upregulated genes in wild type have higher expression levels in the *ΔlreA-*deletion strain **(Figs 4B, 4C and S4)**. 17% (137 out of 785) of the upregulated genes in wild type in red light could not be upregulated anymore **(Fig 4B)**. However, a number of red-light inducible genes (137 genes) detected in wild type were not induced if LreA was absent. On the other hand, 254 genes, which were not induced by red light in wild type, were induced in this strain.

In blue light, 291 genes were upregulated in wild type but, surprisingly, 723 genes were upregulated in the *ΔlreA*-deletion strain **(Fig 4C)**. Only 17% (49 genes out of 291), which were upregulated in wild type in blue light could not be upregulated without LreA. Instead, compared to wild type, 481 genes, which accounted for 67% (481 out of 723) of blue-light inducible genes in the *ΔlreA*-deletion stain, were upregulated additionally. These results demonstrate that LreA has a strong repressive role on gene expression especially in blue light.

### The MAP kinase SakA pathway is the main module for red- and blue light sensing

In red light only 13 genes were upregulated in the *ΔsakA*-deletion strain in comparison to 769 in wild type. Only 1 gene was repressed by red light, in comparison to 220 in wild type **(Fig 4B)**. The 100 genes up- and 15 genes downregulated in the *ΔfphA*-deletion strain did not respond to red-light in the *ΔsakA*-deletion strain.

In blue light, 290 genes were induced in wild type, 97% of which were not induced without SakA. 115 of the upregulated genes encode for enzymes, 60 of which are oxidoreductases. However, 210 genes were upregulated in the *ΔsakA*-deletion strain in blue light. The repression of all 102 blue-light repressed genes in wild type failed in the absence of SakA. In comparison, 80 genes were downregulated in the *ΔsakA*-deletion strain in blue light **(Fig 4C)**. Since the *ΔsakA*-deletion strain is more sensitive to oxidative stress, the observed blue-light dependent and *sakA* independent gene induction could be due to the ROS sensing system.

### Ribosome biogenesis and nitrogen metabolism are adversely regulated

We used the hierarchical cluster analysis next to further characterize the pathways identified in the KEGG analysis. We found that the ribosome biogenesis pathway was strongly downregulated after illumination. In the *ΔfphA*-deletion and the *ΔsakA*-deletion strain, all 24 genes repressed by blue and red light were not repressed anymore **(Fig 4D)**. These results suggest that both, FphA and SakA, appear to be required for the repression by blue light. In contrast, in the *ΔlreA*-deletion strain, these genes still could be repressed by blue light. This is in agreement with the fact that LreA has a repressing function in the dark through direct binding to the promoter of light-regulated genes [28]. LreA leaves the promoter after blue-light illumination [28].

In the KEGG enrichment analysis **(Fig 3D)**, functions of DEGs induced by red light were significantly enriched in the pathway of nitrogen metabolism (p value = 0.012). The genes encode nitrate reductase, nitrite reductase, nitroalkane oxidase and nitronate monooxygenase **(Fig 4E)**. In yeast, nitrate assimilation needs two successive reductions to convert the nitrate to ammonium with nitrate and nitrite reductases. In this study, the genes encoding putative nitrate and nitrite reductases and two genes encoding the nitroalkane oxidase and nitronate monooxygenase able to convert nitroalkane to ammonium were upregulated, which potentially promotes both organic and inorganic nitrogen converted to ammonium. In the meantime, the glutamate dehydrogenase encoding gene was upregulated. Red light is more important for the induction of these genes than far-red and blue light. The induction of these genes was also dependent on FphA and SakA.

### A second layer of light control

We also studied the expression of two other potential genes involved in the *A*. *nidulans* light response, namely the opsin-like protein coding gene *nopA* and the cyptochrome gene, *cryA*. The expression of *nopA* was upregulated in red light in wild type. In the *ΔfphA*-deletion strain and the *ΔsakA*-deletion strain, *nopA* could not be upregulated significantly in red light, but blue light upregulated *nopA* expression when FphA was absent. Interestingly, in the *ΔlreA*-deletion strain, *nopA* was strongly upregulated in both red and blue light **(S6A Fig)**. *cryA* was upregulated in red and blue light in wild type, which was dependent on LreA and SakA. In the *ΔfphA*-deletion strain, red light could not up-regulate *cryA* expression.

The branched phosphorelay system, composed of histidine kinases (HHKs), phosphotransferase YpdA and response regulators, is located upstream of the SakA pathway. The genome of *A*. *nidulans* encodes 15 HHKs including the red-light receptor FphA. In blue and red light, 6 HHK (*tcsB*, *hk2*, *hk-8-1*, *hk-8-2*, *hk-8-3* and *hk-8-5*) encoding genes and the *ypdA* gene were upregulated in wild type and the mutants **(Fig 4F)**. In blue light, 6 HHK-encoding genes (*tcsB*, *hk2*, *hk-8-1*, *hk-8-2*, *hk-8-3* and *hk-8-5*) were more significantly upregulated in the *ΔlreA*-deletion strain than in wild type, but the *ΔfphA*-deletion strain and the *ΔsakA*-deletion strain could not be induced by blue light.

In *N*. *crassa* the DEGs regulated by light were expressed hierarchically and sorted into early and late light-responsive genes [19]. In this study, we identified 21 putative TF encoding genes, which were induced upon red and blue light **(top ten listed in S6B Fig)**. In the *ΔlreA*-deletion strain, the induction of these genes in wild type resembled the one in wild type. However, in the *ΔfphA*- or the *ΔsakA*-deletion strains, most of the TF encoding genes were not upregulated significantly in red light, but in blue light some genes could still be induced.

### The light signaling pathways have functions in the dark

**Fig 5:**
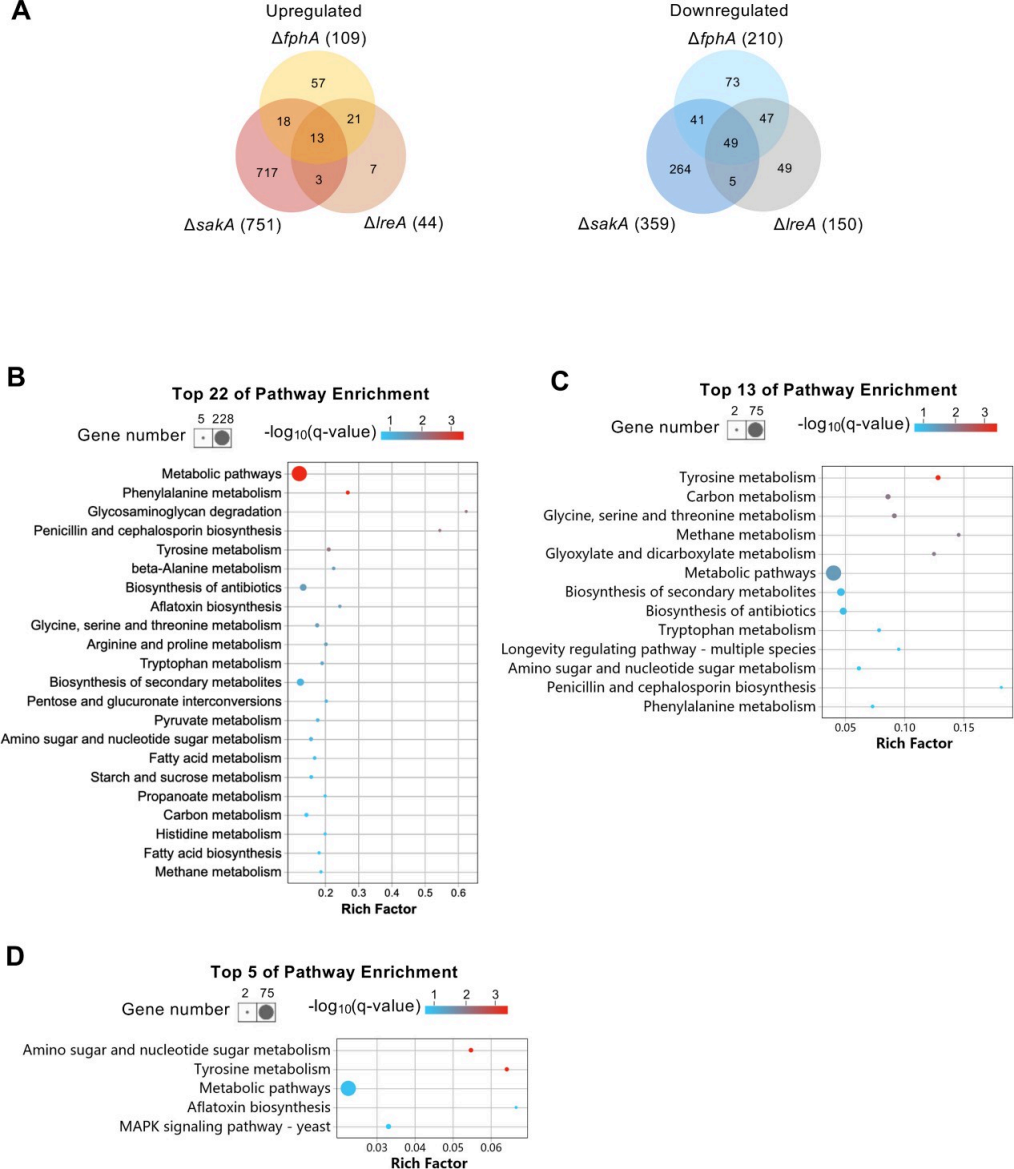
Venn diagram and KEGG pathway enrichment analyses of DEGs identified in the mutants in comparison to wild type in the dark. Venn diagram analyses **(A)** performed with up- and downregulated genes in *ΔsakA*, *ΔfphA*, *ΔlreA* mutants. KEGG pathway analyses conducted with the DEGs identified in *ΔsakA* **(B)**, *ΔfphA* **(C)**, *ΔlreA* **(D),** respectively. The bubble charts were created with significantly enriched pathways (p-value ≤ 0.05). The color of the bubbles indicates the value of -log_10_(q-value) and the q-value represents corrected p-value. Rich factor refers to the quotient of the number of DEGs and the total gene amount in the pathway.

We also analyzed the functions of FphA, LreA and SakA in the dark. In comparison to wild type, 109 genes in the *ΔfphA*-deletion strain were up- and 210 genes downregulated **(Figs 5, S5C and S5D)**. In the *ΔlreA*-deletion strain, only 44 genes were downregulated, but 150 genes were upregulated. Surprisingly, in the *ΔsakA*-deletion strain, 751 genes were upregulated and 359 genes downregulated in the dark in comparison to wild type. In the KEGG pathway analysis performed with all 1110 DEGs, the top 22 enriched pathways include the pathways of amino acid metabolism, carbon metabolism, fatty acid metabolism and biosynthesis of secondary metabolites.

### The blue-light response in the absence of LreA requires cryptochrome and phytochrome

**Fig 6:**
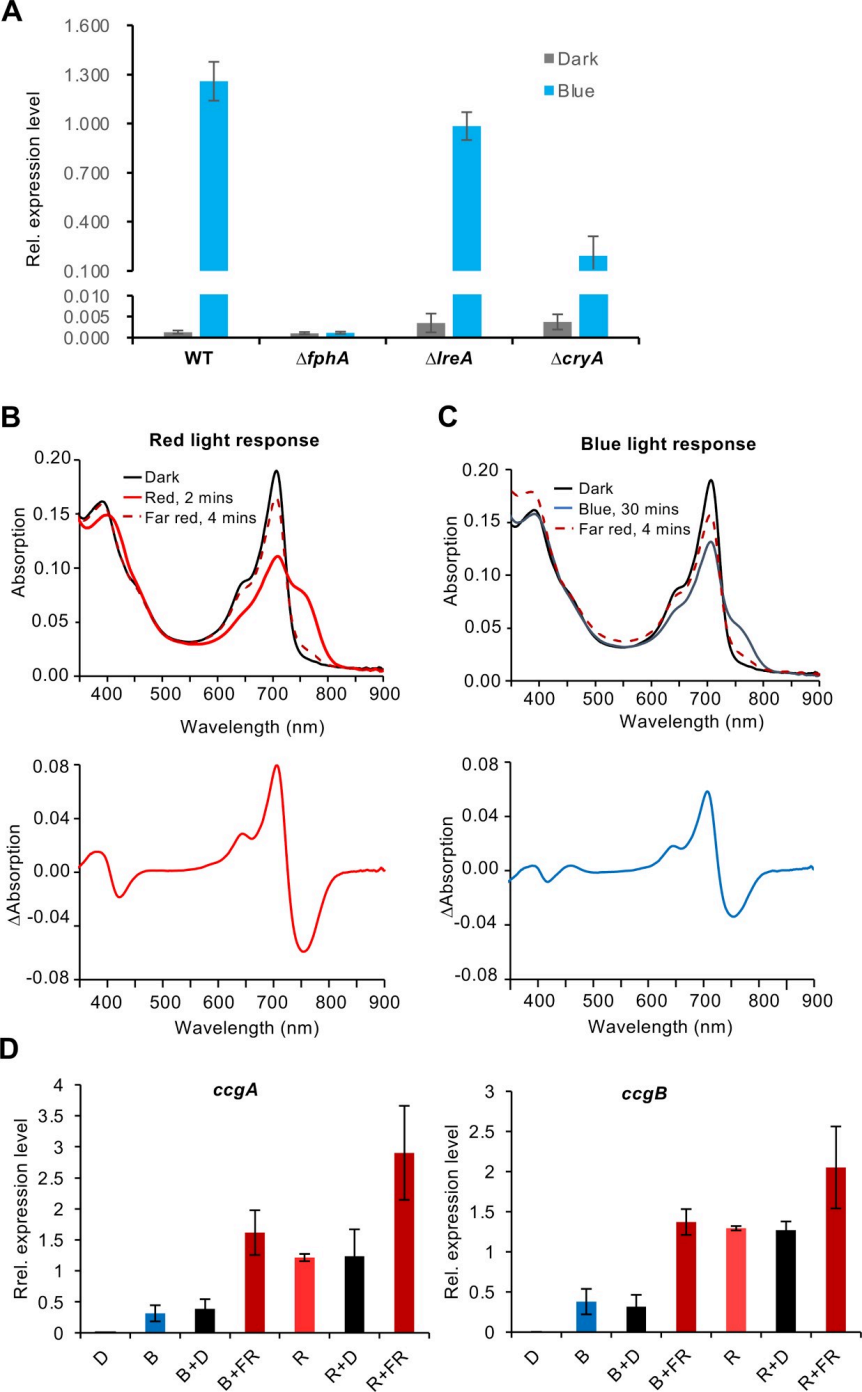
Role of cryptochrome and phytochrome in the blue-light response. **(A)** *ccgB* expression level in wild type, the *ΔfphA-*, the *ΔlreA-* and the *ΔcryA*-deletion strains. The mycelia grown on the surface of the supplemented liquid minimal medium were exposed to blue light 15 min before RNA isolation. The expression of *ccgB* was normalized to the *h2b* gene. The error bar was calculated from three biological replicates. **(B)** Spectral analysis of phytochrome *in vitro*. FphA was purified from *E*. *coli* and a spectrum recorded in the dark, followed by 2 min illumination with red light to convert the P_r_- into the P_fr_ form. The P_fr_ form was illuminated 4 min with far red light to recover the P_r_ form. **(C)** Like in **(B)**, but illumination of the P_r_ form with blue light for 30 min. **(D)** Analysis of the reversibility of the light-induced gene expression. Expression of *ccgA* and *ccgB* in WT (SJR2) in dark, blue, red and blue or red followed by darkness or far-red light. The different phases were 15 min. The error bar was calculated from three biological replicates.

We found that 393 genes were differently expressed upon blue light in wild type, but 82% (324) of them was independent of LreA. This was surprising and raised the question about the involved photoreceptor. We tested a putative role of the blue light photoreceptor CryA on the expression of *ccgB* **(Fig 6A)**. Although the expression level of *ccgB* decreased in blue light when CryA was absent, *ccgB* expression in the *ΔcryA*-deletion strain was still upregulated more than 140-fold in blue light in comparison to the dark. This shows that cryptochrome has some contribution to blue-light regulation but is not essential. Therefore, we tested the role of the phytochrome FphA in the blue-light dependent expression of *ccgB*. Interestingly, in the phytochrome-deletion strain *ccgB* was not induced anymore **(Fig 6A)**, suggesting a main role of phytochrome in blue-light photoinduction. This is supported by the fact that 96% of the blue-light induced genes required SakA. To test whether phytochrome could sense blue light, we purified *A*. *nidulans* phytochrome expressed in *E*. *coli* and performed spectroscopic analyses. Typical phytochrome UV/vis spectra consist of two specific bands, one in the red spectral region around 700 nm (Q-band) and one in the blue spectral region around 400 nm (Soret-band). Activation of phytochrome by red light leads to a conformational change indicated by an increased absorbance in the far-red spectral region around 750 nm **(Fig 6B)**.

To investigate the effect of blue light on FphA, UV/vis absorbance spectrum of heterologous expressed and purified FphA was recorded after irradiation with blue light (450 nm, 30 min) (**Fig 6C)**. The irradiation with blue light resulted in the formation of a shoulder in the far-red spectral region at 754 nm indicating the formation of the active (P far-red) Pfr form. The maximum of the shoulder generated by the irradiation with blue light resembles the maximum of the peak generated by the irradiation with red light, even though the kinetics in case of blue light is much slower and the amplitude lower than with red light. In summary, these results show that phytochrome also senses blue light and probably signals as well through the HOG pathway. However, red light appears to be much more effective given that many more genes are phytochrome-dependent regulated under red-light than under blue-light conditions.

Since the interconversion of the chromatic properties of phytochrome and the reversibility of red-light induced biological responses by far-red light illumination, we tested whether the observed gene inductions could be reversed. To this end, we illuminated mycelia with red or blue light followed by far-red light illumination. However, the transcript abundance both tested genes, *ccgA* and *ccgB*, was not decreased but with far-red light rather increased **(Fig 6D)**.

## Discussion

Light is very important for non-photosynthetic organisms as environmental signal. We found that in *A*. *nidulans* the transcription level of more than 1100 genes changes after 15 min of illumination (red-, far-red and blue light). This is about 10% of the entire genome and much more than what was reported so far. In *A*. *nidulans* about 500 genes were described as light regulated using microarray analyses and 30 min of white light exposure [38]. Although a large proportion of genes were identified in both studies, many were also only found in one study **(S7 Fig)**. This is probably due to the different methods used. In the array analysis 30 min of white light were applied, whereas in our new study individual wavelengths were used. A large number of light-regulated genes were also found in *N*. *crassa*, where 300 genes were under light control (5–240 min white light, microarrays) and in *T*. *harzianum* with 460 (blue light 34 sec followed by 30 min in the dark, RNAseq) genes [19,22]. In *Ustilago maydis* about 300 genes were blue-light regulated and additionally some responded to red or to far-red light [48]. Although the conditions in the different organisms were not identical, the number of regulated genes is comparable. The fact that our RNAseq analyses revealed a significantly higher number of light-regulated genes may be due to the fact that we studied the effect of red-, far-red and blue light separately. This should increase the resolution because combinations of different light qualities could have adverse effects on the transcription of certain genes. In addition, we found that *ccgA* and *conJ* expression level is higher after 15 min illumination than after 30 min. We also know that phosphorylation of SakA occurs already after 5 min of illumination [31]. Hence, the light response is rather quick and probably transient.

The huge impact light has on gene expression raises the question if these changes are of importance under natural conditions, because monochromatic light is rather artificial. However, the spectral composition of light may be different in different habitats, such as under the canopy of plants or in the soil. It was shown that far-red light penetrates deeper into soil and leaf litter than light of shorter wavelengths [49]. Chromatic adaptation of cyanobacteria growing in different zones in a water column resembles this effect [50]. The spectral properties also change during the course of a day and fine-tuning of gene expression is likely, but probably far below the resolution of the experiments under laboratory conditions.

**Fig 7:**
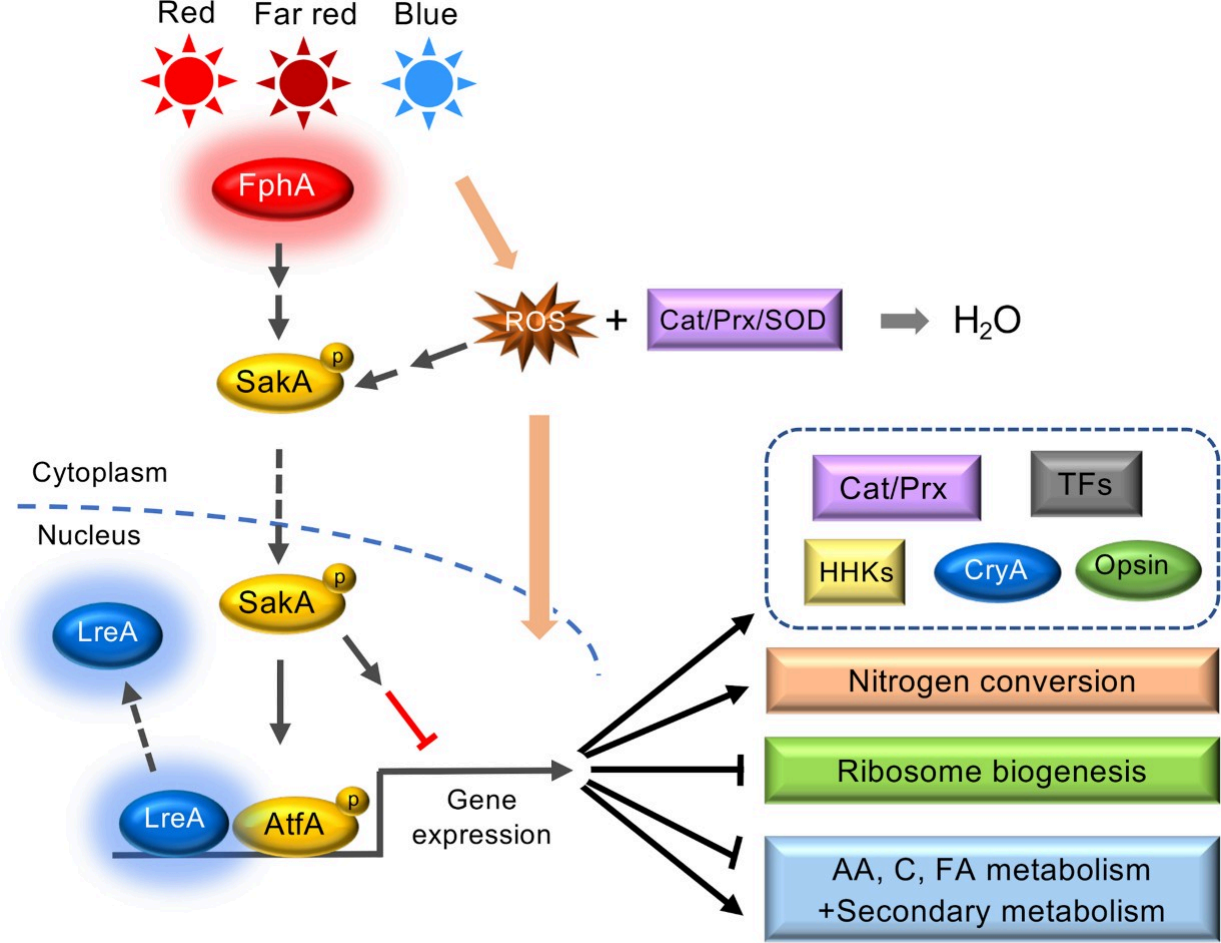
Model for light regulating pathways in *A*. *nidulans*. LreA acts as repressor under blue- and red-light conditions, while FphA senses blue, red and far-red light. Besides LreA and FphA, reactive oxygen species (ROS) may be used as a read-out for illumination with blue-light. For further details refer to the Discussion.

Our analyses revealed several interesting novel aspects **(Fig 7)**. The fact that ribosome biogenesis appears to be downregulated, may suggest a pausing step before massive changes of the metabolome are initiated. This could be a safety step to prevent unnecessary changes upon transient changes of the environmental conditions. In nature, illumination probably changes frequently by e.g. moving branches, without causing immediate drastic changes of other environmental conditions. Hence, a measure for some persistency of environmental changes would be advantageous and may be achieved through a control of the translation efficiency.

Another interesting aspect is the role of phytochrome in blue-light and in far-red light sensing **(Fig 7)**. Already in previous experiments we found that far-red light had a stronger effect on the delay of germination, which is now emphasized by the gene-expression data [40]. In plants, PhyA but not PhyB, C, D and E also responds to far-red light, [51,52]. This phenomenon is not fully understood yet, but it appears that the ratio between the Pr and the Pfr form is important. The photoconversion of phytochrome is never 100% because the absorption spectra of Pr and Pfr overlap. There is also evidence in *Arabidopsis* that phytochrome may play a role in blue-light sensing [53]. We also found that cryptochrome plays a role in blue-light sensing. However, since *A*. *nidulans* CryA has a dual function as light sensor with photolyase activity, the connection to gene expression may be indirect [54]. It is even possible that cryptochrome interacts with the phytochrome system [53,55].

One main characteristic of phytochrome is the interconversion between the different conformations. The conformational changes induced by illumination with red light can be reversed by far-red light illumination. Also at the biological level the response is reversable. In *A*. *nidulans* conidiation is induced by 30 min of red light illumination, and if red-light was followed by 30 min of far-red light illumination and the mycelia incubated further for 24 h in the dark, conidia production was not induced. In contrast, we found no reversability of the red- or blue-light induced expression of *ccgA* and *ccgB*, although it clearly happens *in vitro*. The different results at the gene-expression level and at the biological response may be due to the different timings, given that conidiation takes 24 hours and we measured the transcript abundance after 15 min.

It was also surprising to see that about 100 genes were red-light induced in the absence of phytochrome, but their expression still required SakA. This suggests alternative mechanisms for red-light sensing, which also depend on the HOG pathway. At the moment, it remains an open question if these responses require some kind of photoreceptor proteins or are mediated by mechanisms like the blue-light induced ROS production and secondary effects due to ROS. Indeed, the expression of enzymes involved in ROS detoxification may also be controlled by light as it was shown in *A*. *alternata* [56].

The unraveling of the complex light-regulatory system in fungi remains a challenging task. Besides the gap of the biological meaning of the rather complicated and diverse light-regulatory systems in fungi, one big gap in our knowledge concerns the mechanisms of light regulations. It appears pretty clear that the action of different photoreceptors, probably in concerted action, along with different other regulators is tightly interwoven with metabolic and morphogenetic pathways.

## Supporting information

S1 Fig Red-light dependent gene induction. **(A)** The quality of the LEDs was measured by JAZ-COMBO S/N:JAZA0503 with a QP400-1-VIS-NIR and CC-3-UV-S spectrometer unit by Ocean Optics. **(B)** Expression analysis of *ccgA* and *ccgB* at different time points by real-time PCR. Fresh conidia of the wild type strain (SJR2) were inoculated on the surface of supplemented liquid minimal medium (2% glucose) and cultured for 18 hours at 37°C in the dark. Afterwards, red light (1.7 μmol photons m^-2^ s^-1^) was imposed for 15 min or 30 min before RNA isolation. The expression of *ccgA* and *ccgB* was normalized to the *h2b* gene. The error bar was calculated from three biological replicates.
(PDF)

S2 Fig Luciferase-based reporter assay as described in Fig 1 at different scales. (PDF)

S3 Fig Repression of ribosome biogenesis by light. The encoding genes of the proteins in the green boxes were significantly downregulated in red, blue or far-red light. The figure was created based on the map of ribosome biogenesis for *A*. *nidulans*.
(PDF)

S4 Fig Heatmap of all DEGs identified in all strains in red and blue light in all strains. The colors of heatmap represent log2(fold change) of DEGs. Color scale, -3≤log2 (fold change) ≤3. In cluster 1, 2, 6 and 8, most of the genes are up- or downregulated in wild type (WT) and the *ΔlreA*-deletion strain upon red and blue light did not respond to light anymore in the *ΔfphA*- and the *ΔsakA*-deletion strains. Most of the genes in cluster 3 and 4 are differentially expressed in the *ΔsakA*-deletion strain upon red light and especially upon blue light exposure. Cluster 5 is a gene set of DEGs in the *ΔfphA*-deletion strain upon red light. In cluster 7 and 9, most of the genes could not be induced by blue light in wild type but were upregulated in the *ΔfphA*-deletion strain upon red light. Far-red light responsive genes were not included, because we did not analyze them in the mutants.
(PDF)

S5 Fig Validation of the expression of some genes. **(A)** Red light induction of AN5401 was independent of FphA. **(B)** Blue light induction of AN1457 was independent of FphA. **(C)** Expression of AN2530 was downregulated in the *ΔfphA*-deletion strain. **(D)** Expression of AN2680 was upregulated in *ΔlreA-*deletion strain. Red or blue light was imposed for 15 min. The expression level was normalized to *h2b*. The error bar was calculated from three biological replicates. Significant differences were calculated using the two-sample *t*-test (*P<0.05, **P<0.01 and ***P<0.001).
(PDF)

S6 Fig **Heatmap of *nopA*, *cryA* (A), top 10 light-regulated transcription factors (B) upon red and blue light in different strains.** The colors of heatmap represent log2(fold change) of DEGs. Range of log2(fold change) is indicated in the color bar.
(PDF)

S7 Fig Venn diagram analysis of DEGs identified in this and a previous study. The circle on the left represents the number of the DEGs identified in wild type after 30 min white light with the microarray analysis by Ruger-Herreros et al., 2011. The right circle represents the number of the DEGs identified after 15 min red, far-red or blue light exposure in this study.
(PDF)

S1 Movie Blue light induces transient increases in reactive oxygen species (ROS) in *Aspergillus nidulans*. Hyphae were illuminated with blue light and ROS visualized with CellROX Orange. The movie shows a time-course of 30 min with pictures taken every 20 sec.
(MP4)

S1 Table List of differentially expressed genes in different strains and light conditions as indicated. (XLSX)

S2 Table Number of genes in each GO term in Fig 3C. (XLS)

S3 Table Gene list corresponding to KEGG pathways in Fig 3D. (XLS)
