# Supplementary material for: Genome-wide analyses of light-regulated genes in Aspergillus nidulans reveal a complex interplay between different photoreceptors and novel photoreceptor functions
Source: PLoS Genet. 2021 Oct 22;17(10):e1009845. doi: 10.1371/journal.pgen.1009845 (PMC8535378; doi:10.1371/journal.pgen.1009845)
Supplement: S1 Fig — (A) The quality of the LEDs was measured by JAZ-COMBO S/N:JAZA0503 with a QP400-1-VIS-NIR and CC-3-UV-S spectrometer unit by Ocean Optics. (B) Expression analysis of ccgA and ccgB at different time points by real-time PCR. Fresh conidia of the wild type strain (SJR2) were inoculated on the surface of supplemented liquid minimal medium (2% glucose) and cultured for 18 hours at 37°C in the dark. Afterwards, red light (1.7 μmol photons m-2 s-1) was imposed for 15 min or 30 min before RNA isolation. The expression of ccgA and ccgB was normalized to the h2b gene. The error bar was calculated from three biological replicates. (PDF) [file pgen.1009845.s001.pdf]

Supporting information

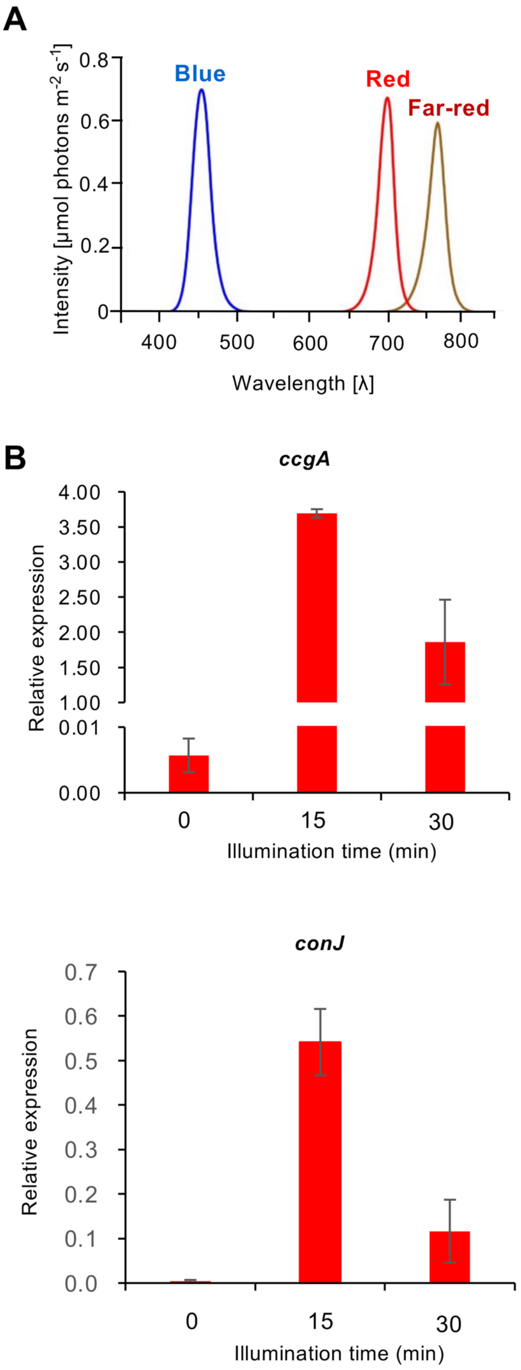

Fig. S1: Red-light dependent gene induction. (A) The quality of the LEDs was measured by JAZ-COMBO S/N:JAZA0503 with a QP400-1-VIS-NIR and CC-3-UV-S spectrometer unit by Ocean Optics. **(B)** Expression analysis of *ccgA* and *ccgB* at different time points by real-time PCR. Fresh conidia of the wild type strain (SJR2) were inoculated on the surface of supplemented liquid minimal medium (2 % glucose) and cultured for 18 hours at 37 °C in the dark. Afterwards, red light (1.7 µmol photons m^{-2} s^{-1}) was imposed for 15 min or 30 min before RNA isolation. The expression of *ccgA* and *ccgB* was normalized to the *h2b* gene. The error bar was calculated from three biological replicates.
