# Supplementary material for: Genome-wide analyses of light-regulated genes in Aspergillus nidulans reveal a complex interplay between different photoreceptors and novel photoreceptor functions
Source: PLoS Genet. 2021 Oct 22;17(10):e1009845. doi: 10.1371/journal.pgen.1009845 (PMC8535378; doi:10.1371/journal.pgen.1009845)
Supplement: S2 Fig — (PDF) [file pgen.1009845.s002.pdf]

Supporting information

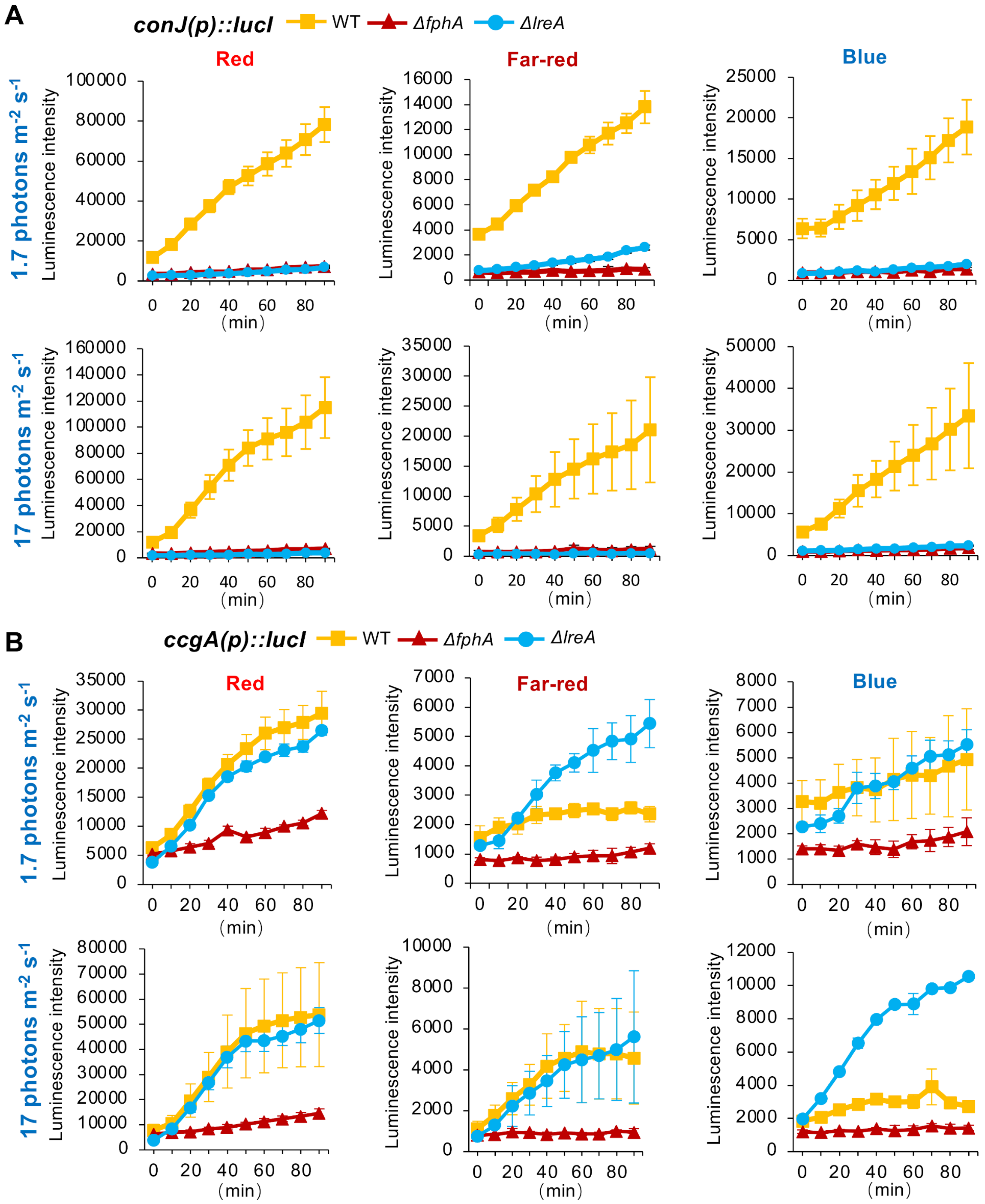

Fig. S2: Luciferase-based reporter assay as described in Fig. 1 at different scales.
