# Supplementary material for: Genome-wide analyses of light-regulated genes in Aspergillus nidulans reveal a complex interplay between different photoreceptors and novel photoreceptor functions
Source: PLoS Genet. 2021 Oct 22;17(10):e1009845. doi: 10.1371/journal.pgen.1009845 (PMC8535378; doi:10.1371/journal.pgen.1009845)
Supplement: S3 Fig — The encoding genes of the proteins in the green boxes were significantly downregulated in red, blue or far-red light. The figure was created based on the map of ribosome biogenesis for A. nidulans. (PDF) [file pgen.1009845.s003.pdf]

# Supporting information

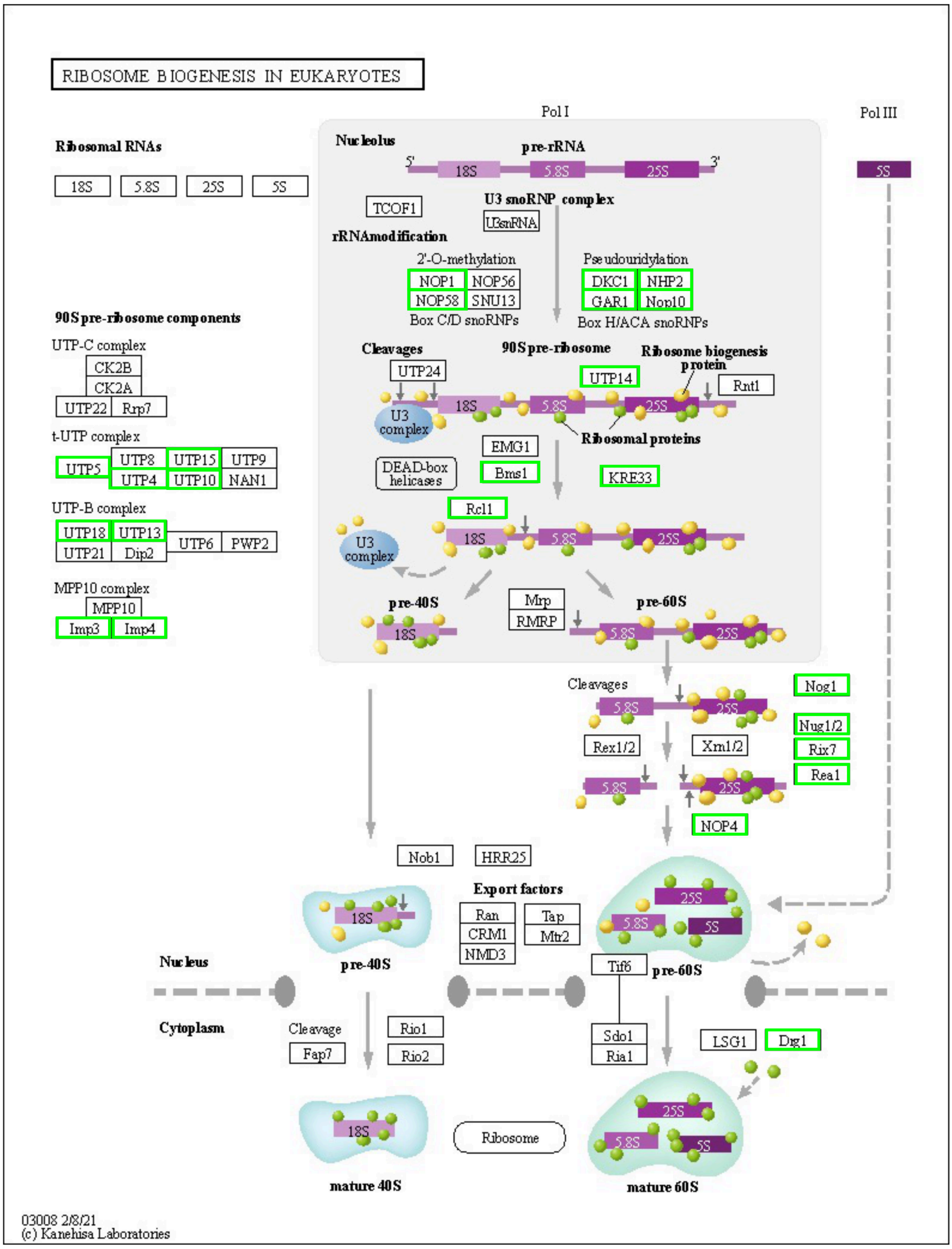


**Fig. S3: Repression of ribosome biogenesis by light.** The encoding genes of the proteins in the green boxes were significantly downregulated in red, blue or far-red light. The figure was created based on the map of ribosome biogenesis for *A. nidulans*.
