# Supplementary material for: Genome-wide analyses of light-regulated genes in Aspergillus nidulans reveal a complex interplay between different photoreceptors and novel photoreceptor functions
Source: PLoS Genet. 2021 Oct 22;17(10):e1009845. doi: 10.1371/journal.pgen.1009845 (PMC8535378; doi:10.1371/journal.pgen.1009845)
Supplement: S4 Fig — The colors of heatmap represent log2(fold change) of DEGs. Color scale, -3≤log2 (fold change) ≤3. In cluster 1, 2, 6 and 8, most of the genes are up- or downregulated in wild type (WT) and the ΔlreA-deletion strain upon red and blue light did not respond to light anymore in the ΔfphA- and the ΔsakA-deletion strains. Most of the genes in cluster 3 and 4 are differentially expressed in the ΔsakA-deletion strain upon red light and especially upon blue light exposure. Cluster 5 is a gene set of DEGs in the ΔfphA-deletion strain upon red light. In cluster 7 and 9, most of the genes could not be induced by blue light in wild type but were upregulated in the ΔfphA-deletion strain upon red light. Far-red light responsive genes were not included, because we did not analyze them in the mutants. (PDF) [file pgen.1009845.s004.pdf]

Supporting information

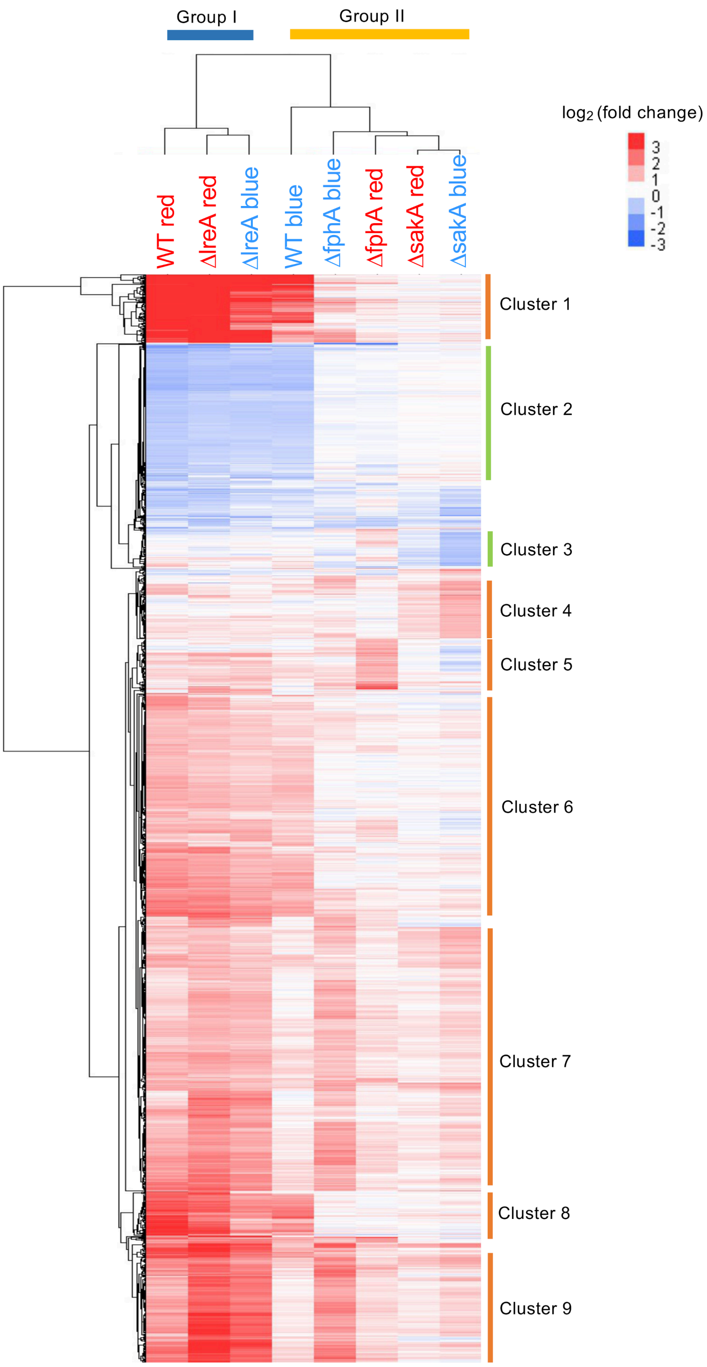

Fig. S4: Heatmap of all DEGs identified in all strains in red and blue light in all strains. The colors of heatmap represent log2(fold change) of DEGs. Color scale, -3≤log2(fold change)≤3. In cluster 1, 2, 6 and 8, most of the genes are up- or downregulated in wild type (WT) and the *ΔlreA*-deletion strain upon red and blue light did not respond to light anymore in the *ΔfphA*- and the *ΔsakA*-deletion strains. Most of the genes in cluster 3 and 4 are differentially expressed in the Δ*sakA*-deletion strain upon red light and especially upon blue light exposure. Cluster 5 is a gene set of DEGs in the *ΔfphA*-strain upon red light. In cluster 7 and 9, most of the genes could not be induced by blue light in wild type but were upregulated in the Δ*fphA*-deletion strain upon red light. Far-red light responsive genes were not included, because we did not analyze them in the mutants.
