# Supplementary material for: Genome-wide analyses of light-regulated genes in Aspergillus nidulans reveal a complex interplay between different photoreceptors and novel photoreceptor functions
Source: PLoS Genet. 2021 Oct 22;17(10):e1009845. doi: 10.1371/journal.pgen.1009845 (PMC8535378; doi:10.1371/journal.pgen.1009845)
Supplement: S5 Fig — (A) Red light induction of AN5401 was independent of FphA. (B) Blue light induction of AN1457 was independent of FphA. (C) Expression of AN2530 was downregulated in the ΔfphA-deletion strain. (D) Expression of AN2680 was upregulated in ΔlreA-deletion strain. Red or blue light was imposed for 15 min. The expression level was normalized to h2b. The error bar was calculated from three biological replicates. Significant differences were calculated using the two-sample t-test (*P<0.05, **P<0.01 and ***P<0.001). (PDF) [file pgen.1009845.s005.pdf]

## Supporting information

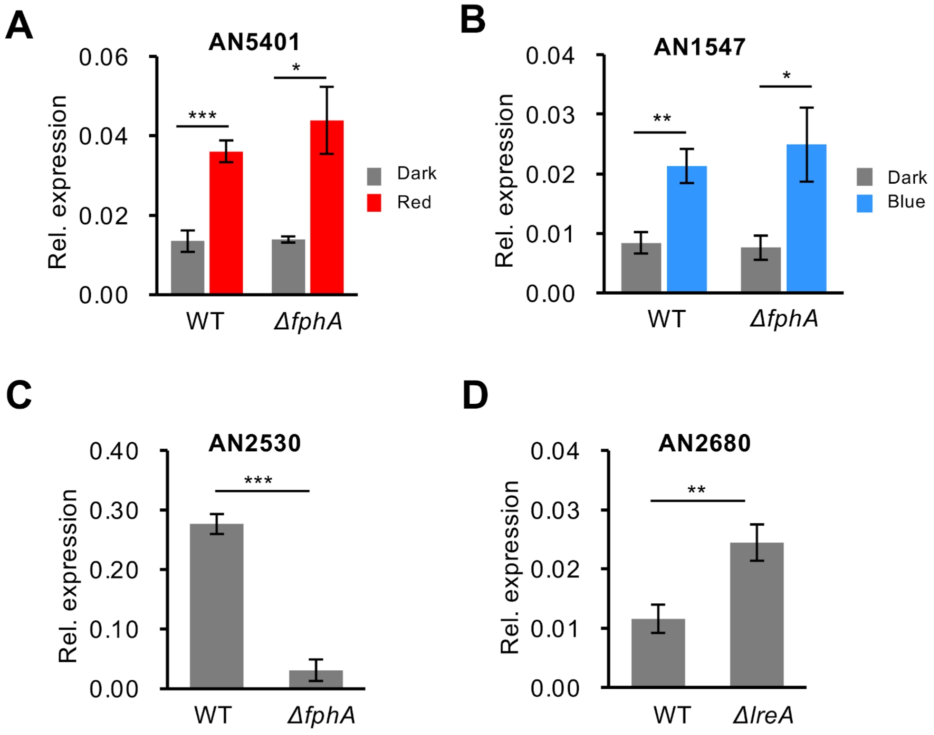


**Fig. S5: Validation of the expression of some genes. (A)** Red light induction of AN5401 was independent of FphA. **(B)** Blue light induction of AN1457 was independent of FphA. **(C)** Expression of AN2530 was downregulated in the *fphA*-deletion strain. **(D)** Expression of AN2680 was upregulated in *lreA*-deletion strain. Red or blue light was imposed for 15 min. The expression level was normalized to *h2b*. The error bar was calculated from three biological replicates. Significant differences were calculated using the two-sample *t*-test (**P*<0.05, ***P*<0.01 and ****P*<0.001).
