# Supplementary material for: Genome-wide analyses of light-regulated genes in Aspergillus nidulans reveal a complex interplay between different photoreceptors and novel photoreceptor functions
Source: PLoS Genet. 2021 Oct 22;17(10):e1009845. doi: 10.1371/journal.pgen.1009845 (PMC8535378; doi:10.1371/journal.pgen.1009845)
Supplement: S6 Fig — Heatmap of nopA, cryA (A), top 10 light-regulated transcription factors (B) upon red and blue light in different strains. The colors of heatmap represent log2(fold change) of DEGs. Range of log2(fold change) is indicated in the color bar. (PDF) [file pgen.1009845.s006.pdf]

Supporting information

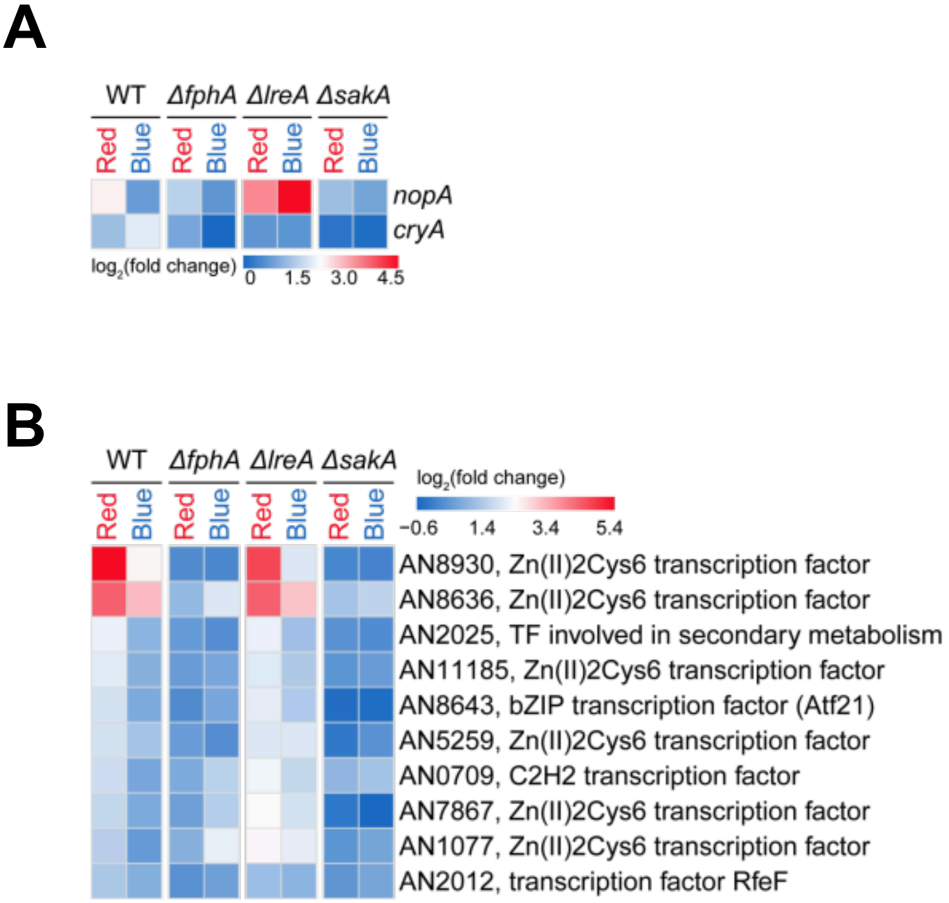

Fig. S6: Heatmap of *nopA*, *cryA* (A), top 10 light-regulated transcription factors (B) upon red and blue light in different strains. The colors of heatmap represent log2(fold change) of DEGs. Range of log2(fold change) is indicated in the color bar.
