# Supplementary material for: Genome-wide analyses of light-regulated genes in Aspergillus nidulans reveal a complex interplay between different photoreceptors and novel photoreceptor functions
Source: PLoS Genet. 2021 Oct 22;17(10):e1009845. doi: 10.1371/journal.pgen.1009845 (PMC8535378; doi:10.1371/journal.pgen.1009845)
Supplement: S7 Fig — The circle on the left represents the number of the DEGs identified in wild type after 30 min white light with the microarray analysis by Ruger-Herreros et al., 2011. The right circle represents the number of the DEGs identified after 15 min red, far-red or blue light exposure in this study. (PDF) [file pgen.1009845.s007.pdf]

## Supporting information

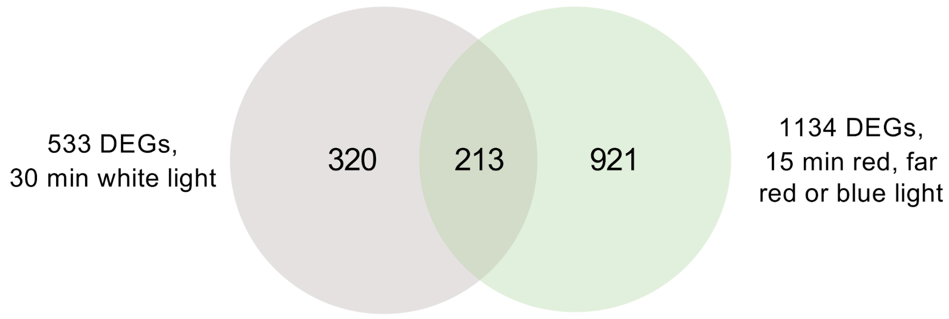


**Fig. S7: Venn diagram analysis of DEGs identified in this and a previous study.** The circle on the left represents the number of the DEGs identified in wild type after 30 min white light with the microarray analysis by Ruger-Herreros et al., 2011. The right circle represents the number of the DEGs identified after 15 min red, far-red or blue light exposure in this study.
